# Lie Classification and Symmetry-Preserving Reduced-Order Modeling of Nonlinear Electrostatic MEMS

**DOI:** 10.3390/mi17091095

**Published:** 2026-09-17

**Authors:** Mario Versaci, Francesco Carlo Morabito

**Affiliations:** Dipartimento DICEAM, Università Mediterranea degli Studi di Reggio Calabria, Via R. Zehebder s.n.c, I-89122 Reggio Calabria, Italy; morabito@unirc.it

**Keywords:** electrostatic MEMS, lie symmetry analysis, reduced-order modeling, symmetry-preserving model reduction, physics-informed modeling, pull-in instability, fringing fields, digital twins

## Abstract

High-fidelity continuum models of electrostatically actuated MEMS accurately capture distributed electromechanical interactions but are computationally expensive for repeated simulation, optimization, and real-time applications. This work develops a physics-informed reduced-order modeling framework based on Lie symmetry classification for a nonlinear electrostatic MEMS microplate governed by a fourth-order integro-partial differential equation. The continuum model is recast as an extended canonical system separating local differential operators from nonlocal stretching and capacitive contributions. Lie group classification of the complete boundary value problem shows that the electrostatic singularity, constitutive coefficients, fixed geometry, and clamped boundary conditions suppress nontrivial continuous spatial symmetries in the generic bounded problem, while time translation survives only in the autonomous subclass. The discrete reflection invariances of the centered rectangular device are treated separately to identify invariant functional subspaces for Galerkin projection. A symmetry-preserving reduced-order model is then constructed in the even–even subspace, retaining bending, geometric stretching, pre-stress, capacitive feedback, dielectric inhomogeneity, and fringing field effects. Numerical verification against the high-fidelity continuum model shows close agreement with the FOM for static and transient responses while preserving reflection symmetry and remaining robust under parameter variations. For the nominal configuration, the monomodal Lie-ROM predicts a pull-in voltage of 127.73V versus 128.21V for the FOM, corresponding to an absolute relative error of 0.37% and a signed error of −0.37%. The monomodal formulation substantially reduces computational cost and consistently outperforms a classical lumped-parameter approximation, providing an interpretable and efficient basis for parametric analysis, design optimization, control-oriented modeling, and future digital twin applications.

## 1. Introduction

**Figure 1:**
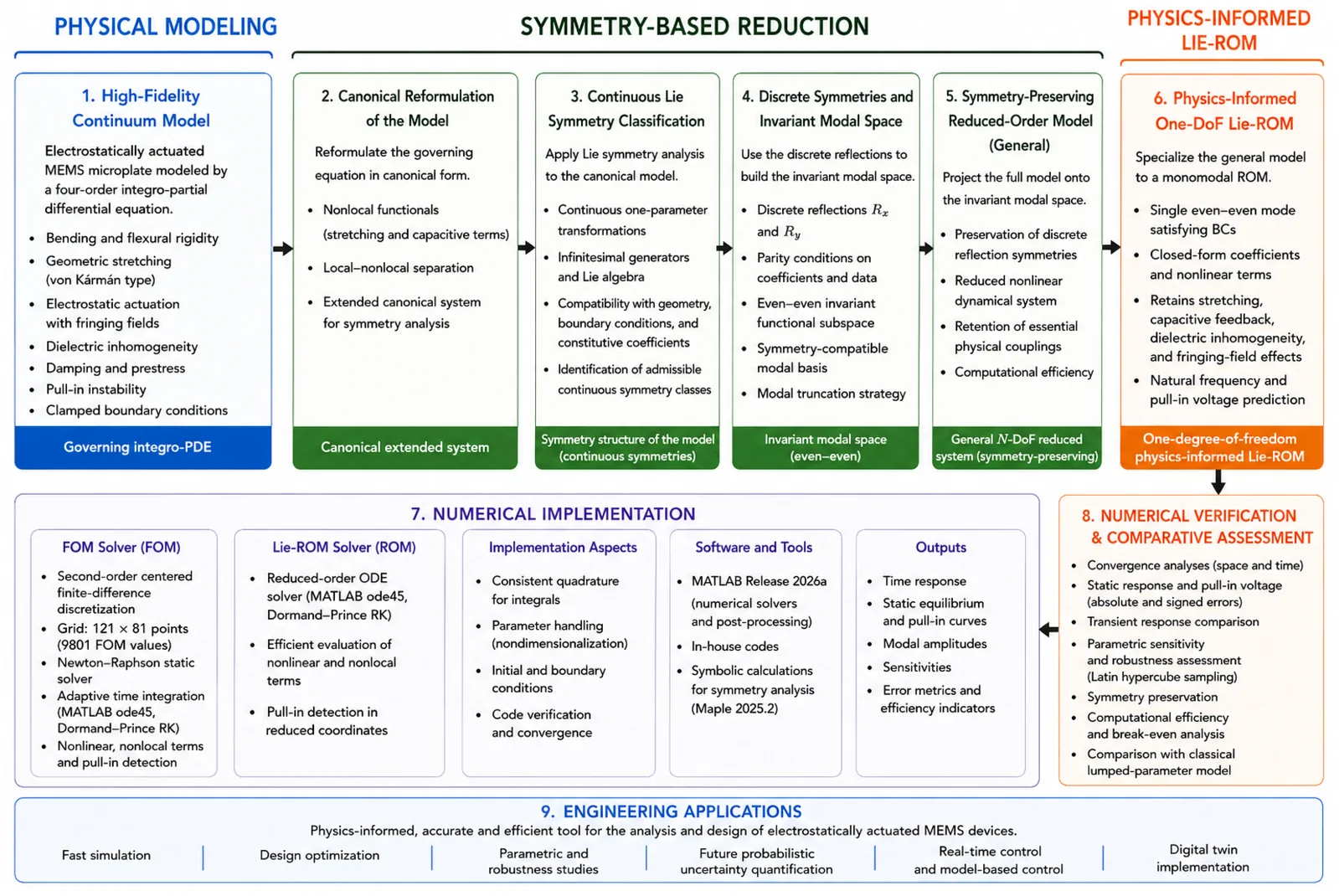
Workflow of the proposed Lie symmetry-preserving reduced-order modeling framework for nonlinear electrostatically actuated MEMS.

Electrostatic MEMS are widely employed in actuators, sensors, micromirrors, resonators, microvalves, and signal processing devices [1,2,3,4,5,6] owing to their compatibility with microfabrication technologies, low power consumption, and potential for on-chip integration [7]. Their design requires accurate modeling of the nonlinear electrostatic force $f_{\mathrm{es}}$ [8,9,10], structural deformability [11,12], fringing field effects $E_{\mathrm{ff}}$ [13,14,15], and pull-in instability [16,17,18,19,20,21,22,23]. Depending on the required accuracy and computational cost [24,25,26,27], models range from high-fidelity continuum formulations [28,29] to lumped-parameter descriptions [30]. Continuum PDE models provide a high-fidelity description when the spatial distributions of deformation and the electrostatic field E cannot be neglected [31,32]. They can account for flexural stiffness [32], in-plane stress [33], geometric stretching [34], damping [35], spatially varying dielectric properties [36,37], and $E_{\mathrm{ff}}$ effects [38]. This fidelity entails substantial computational cost [39], particularly for repeated analyses involving parameter variations [40], system identification [40], control design [41], and optimization [42]. Conversely, lumped models with one or only a few DoFs are computationally efficient but may neglect the spatial distribution of the physical fields [43] and oversimplify the electromechanical coupling [44]; near pull-in, this can lead to significant errors in the predicted deflection, natural frequency, and critical voltage [45,46]. Reduced-order modeling provides a compromise between accuracy and computational efficiency [47,48]. Although classical modal methods [48], proper orthogonal decomposition [49], balanced truncation [50], and data-driven approaches can substantially reduce the number of DoFs [49,50], their effectiveness relies on a high-fidelity reference formulation capable of retaining the dominant electromechanical mechanisms. In this work, the reference model is the fourth-order nonlinear dynamic formulation proposed and analyzed in [51,52], derived from classical plate theory and extended to include $E_{\mathrm{ff}}$ effects according to the Pelesko–Driscoll approach [53,54]. It incorporates microplate bending, nonlocal geometric stretching, capacitive feedback, spatially distributed dielectric properties, and $E_{\mathrm{ff}}$ effects, with established results on existence, uniqueness, and regularity. While Campanato’s Near Operator Theory provides the analytical foundation for its well-posedness and regularity [51], the present work addresses the complementary problem of structure-preserving model reduction through Lie symmetry analysis. The proposed physics-informed methodology is constructed directly from the high-fidelity continuum formulation [51] with the aim of reducing the number of DoF while preserving its essential physical and mathematical structure. The role of Lie symmetry analysis in this work differs from that of a classical Lie similarity reduction [53]. The complete boundary value problem includes a fixed bounded geometry, clamped boundary conditions, nonlocal electromechanical couplings, constitutive heterogeneity, and a fixed electrostatic contact manifold. Lie-group classification is used to identify the continuous transformations compatible with all these constraints. For the generic bounded configuration, nontrivial continuous spatial generators are excluded, while time translation survives only in the autonomous case; this negative result is structurally relevant because it identifies the continuous reductions incompatible with the physical problem. In this sense, the Lie analysis does not replace the Galerkin projection and is not claimed to produce a different projection once the invariant subspace has been prescribed. Its contribution is upstream: it establishes, from the complete boundary-value problem rather than by prior assumption, which symmetry restrictions are admissible and under which physical and constitutive conditions they remain valid. The bounded rectangular device may retain discrete reflection symmetries when the constitutive coefficients and initial conditions satisfy the required parity conditions. These invariances define functional subspaces used to constrain the subsequent Galerkin projection. The proposed methodology combines continuous Lie classification with invariant subspace construction based on discrete symmetry, rather than performing a classical Lie similarity reduction. Accordingly, the term Lie-ROM denotes a reduced-order model constructed from the systematic classification of the continuous and discrete symmetries compatible with the complete boundary value problem. The main contributions are as follows:

(i) Canonical reformulation of the nonlinear continuum model, providing a structured representation of the coupled electromechanical mechanisms suitable for systematic parametric analysis.
(ii) A Lie group classification of the complete nonlinear electromechanical boundary value problem, identifying the continuous transformations compatible with the electrostatic singularity, nonlocal couplings, constitutive coefficients, fixed geometry, and clamped boundary conditions while demonstrating the suppression of nontrivial continuous spatial symmetries in the generic bounded configuration.
(iii) Identification of the remaining discrete reflection invariances and construction of the corresponding invariant modal subspaces, providing a mathematically consistent basis for symmetry-preserving Galerkin model reduction.
(iv) Specialization of the proposed framework into a physics-informed one-degree-of-freedom Lie-ROM, providing a low-cost and interpretable model suitable for fast simulation, design optimization, parametric studies, and model-based control.
(v) Comprehensive numerical verification and comparative assessment demonstrating accuracy, robustness, symmetry preservation, and computational efficiency. The computational efficiency of the proposed methodology supports its potential for fast simulation, control-oriented applications, and future digital twin implementations of electrostatically actuated MEMS devices. The proposed methodology is assessed through extensive numerical comparisons among the reference continuum model [51], the proposed Lie-ROM, and a classical lumped-parameter model based on the nonlinear mass–spring–damper formulation with parallel-plate electrostatic actuation reported in [55]. All three formulations refer to the same fully clamped rectangular MEMS microplate made of phosphorus-doped low-pressure chemical vapor-deposited (LPCVD) polysilicon, with equivalent parameters derived from its physical and geometric properties. This reference configuration is also consistent with the application-oriented context of the Fa.Per.M.E. project, funded by the Italian Ministry of Health, within which the present research was partially developed. The resulting workflow is summarized in Figure 1.

The remainder of this paper is organized as follows: Section 2, Section 3 and Section 4 describe the MEMS device, the reference model [51], and its canonical reformulation; Section 5 and Section 6 develop the Lie symmetry analysis and the resulting symmetry-preserving reduced-order framework, including its physics-informed single-DoF specialization; Section 7 describes the numerical implementation, while Section 8 provides extensive numerical verification and comparative assessment against the reference continuum model [51] and a classical lumped-parameter model, including convergence, static and transient responses, parametric sensitivity, computational efficiency, and symmetry preservation; finally, Section 9 summarizes the main findings and future research directions.

## 2. The Device Under Study

**Figure 2:**
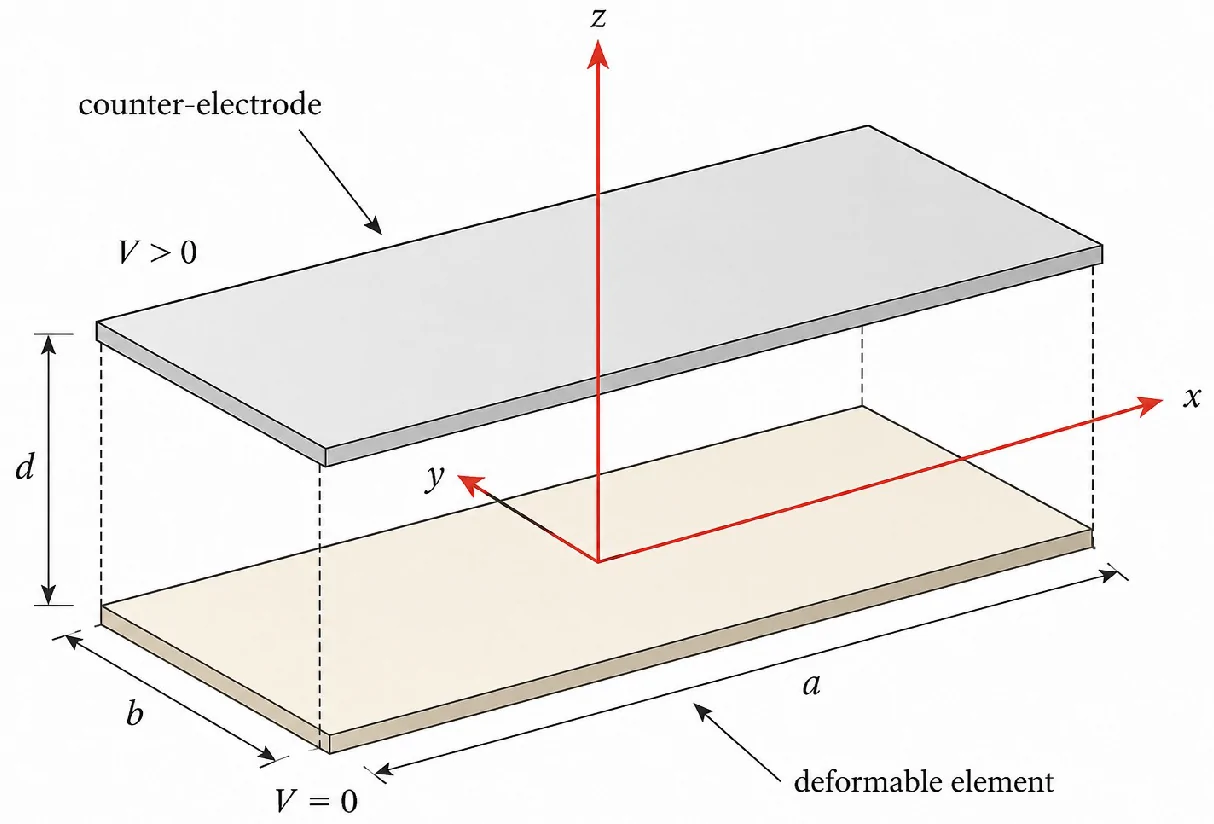
The electrostatic MEMS device under study.

The MEMS consists of two parallel conductive electrodes separated by an initial gap *d*. The upper electrode is rigid and maintained at the prescribed voltage V(t), where *t* denotes time, whereas the lower electrode is a fully clamped rectangular microplate made of phosphorus-doped LPCVD polysilicon and occupying the bounded domain $\Omega \subset R^{2}$. The applied voltage generates an electrostatic field E(t) and the associated distributed electrostatic pressure, inducing the transverse displacement w(x,t). The normalized displacement satisfies 0≤w(x,t)<1, while w(x,t)=1 corresponds to the ideal pull-in configuration. The mechanical behavior is described by the Kirchhoff–Love plate theory [56], accounting for flexural rigidity and geometric stretching, whereas the electrostatic model includes dielectric inhomogeneity, deformation-dependent capacitive feedback, and $E_{\mathrm{ff}}\left(t\right)$ effects. The same physical device is adopted for the continuum model, the proposed Lie-ROM, and the lumped model considered in [55], so that the three formulations share the same reference physical device and parameter set while differing in their level of spatial resolution and in the electromechanical mechanisms retained by the corresponding reduced descriptions. The device is illustrated in Figure 2. The dimensional variables are normalized according to [51]; the electrostatic actuation is represented by the dimensionless parameter ${\left(\lambda \left(t\right)\right)}^{2}$, the pre-stress by the coefficient γ, and the damping level by the modal damping ratio ζ. The reference dimensional and dimensionless parameters are summarized in Table 1 (where |Ω|=ab=0.6667 denotes the dimensionless plate area; therefore, for d/h=1, the nominal stretching coefficient is $\beta_{0}=9.00$), while the corresponding normalization constants are reported in Table A1.

For the adopted reference device, the characteristic voltage is $V_{b}=5.049\;V$, so that the dimensionless actuation parameter is $\lambda =V/V_{b}$. The capacitive feedback coefficient χ (see the capacitive electrical model in [55]) and the $E_{\mathrm{ff}}$ coefficient $\delta_{0}$ are dimensionless phenomenological parameters of the electromechanical model rather than intrinsic material properties of doped polysilicon. The reference values χ=0.05 and $\delta_{0}=0.02$ are adopted as representative numerical benchmarks, with χ quantifying the nonlocal electrostatic feedback associated with deformation-dependent capacitance and $\delta_{0}$ weighting the additional electrostatic contribution due to $E_{\mathrm{ff}}$ and local slope effects. Their influence is assessed over the ranges 0≤χ≤0.15 and $0\leq \delta_{0}\leq 0.10$. In the nominal homogeneous and autonomous configuration, $\delta \left(x,t\right)=\delta_{0}=0.02$, while spatially or temporally varying distributions may be considered provided that the assumed regularity and parity conditions are preserved. Accordingly, χ and $\delta_{0}$ characterize the selected numerical benchmark and should not be interpreted as universal material constants.

## 3. The Continuous Dynamic Model

The original governing equation can be written as [51]

(1) $$\begin{array}{c} \begin{array}{c} K_{1}\left(x,t\right)\Delta^{2}w\left(x,t\right)+c\left(x,t\right)w_{t}\left(x,t\right)+w_{tt}\left(x,t\right)=\\ =\left[\beta \int_{\Omega }{\left|\nabla w\left(x,t\right)\right|}^{2}\;dx+\gamma \right]\Delta w\left(x,t\right)+\\ +\;\frac{{\left(\lambda \left(t\right)\right)}^{2}p\left(x\right)}{{\left(1-w\left(x,t\right)\right)}^{2}{\left[1+\chi \int_{\Omega }\frac{dx}{1-w(x,t)}\right]}^{2}}+\underset{F_{\mathrm{ff}}}{\underset{︸}{\frac{{\left(\lambda \left(t\right)\right)}^{2}\delta \left(x,t\right){\left|\nabla w\left(x,t\right)\right|}^{2}}{{\left(1-w\left(x,t\right)\right)}^{2}}}}, \end{array} \end{array}$$

where $F_{\mathrm{ff}}$ represents the $E_{\mathrm{ff}}$ contribution with w=0, $w_{n}=0$, x∈∂Ω and $w\left(x,0\right)\;=\;w_{0}\left(x\right)$, $w_{t}\left(x,0\right)=v_{0}\left(x\right)$. Although (1) accurately describes the nonlinear electromechanical behavior of the device, its integro-differential structure is not directly suited to reduced-order modeling because geometric stretching and capacitive feedback involve nonlocal integral operators. The displacement field w(x,t) is assumed sufficiently regular for all bending, inertial, electrostatic, stretching, and $E_{\mathrm{ff}}$ contributions to be well defined. Moreover, the analysis is restricted to the pre-contact regime, w(x,t)<1, ensuring electrode separation and the finiteness of the electrostatic force and associated nonlocal operators. Mathematically, the previous engineering assumptions are expressed by requiring the displacement field to belong to the functional space

(2) $$w\left(x,t\right)\in C^{2}\;\left(\left[0,T\right];L^{2}\left(\Omega \right)\right)\cap C^{1}\;\left(\left[0,T\right];H_{0}^{2}\left(\Omega \right)\right)\cap C\;\left(\left[0,T\right];H^{4}\left(\Omega \right)\cap H_{0}^{2}\left(\Omega \right)\right).$$

The material and electromechanical parameters entering the governing equation are assumed to remain bounded over the spatial domain and the time interval of interest, consistently with the normal operating regime of the device. In particular, the mechanical damping, effective bending stiffness, $E_{\mathrm{ff}}$ correction, and dielectric distribution remain finite, while the actuation voltage varies continuously in time. These assumptions exclude unphysical singularities and ensure that all local and nonlocal electromechanical contributions are well defined. Accordingly, the regularity conditions are $c,\delta ,K_{1}\in L^{\infty }\;\left(\Omega \times (0,T)\right)$, $p\in L^{\infty }\left(\Omega \right)$, λ∈C([0,T]), which provide the functional framework for (1). The physical interpretation of the operators, coefficients, and nonlinear terms entering the governing model is summarized in Table 2.

## 4. Canonical Reformulation of the Continuous Dynamic Model

To separate the nonlocal electromechanical couplings associated with geometric stretching and capacitive feedback from the local differential operators, we introduce the two global functionals,

(3) $$I_{1}\left[w\right]\left(t\right)=\int_{\Omega }{\left|\nabla w\left(x,t\right)\right|}^{2}\;dx,\;I_{2}\left[w\right]\left(t\right)=\int_{\Omega }\frac{1}{1-w(x,t)}\;dx,$$

which replace the integral operators with global variables. This makes the model suitable for Lie symmetry analysis and for naturally entering the reduced-order formulation while preserving the dominant distributed electromechanical mechanisms at significantly lower computational cost. The canonical reformulation is established by the following results, for which proof sketches are reported in Appendix B.

**Lemma 1**  (Well-posedness of the nonlocal functionals)**.** *Assume that the regularity condition *(2)* holds and that there exists a constant ε>0 such that w(x,t)≤1−ε a.e. in Ω×[0,T]. Then, the nonlocal functionals *(3)* are well defined ∀t∈[0,T]. Moreover, under *(2)*, $I_{1}\left[w\right]\left(t\right),\;I_{2}\left[w\right]\left(t\right)\in C\left(\left[0,T\right]\right)$, and as such constitute admissible auxiliary global variables for the canonical reformulation of the governing electromechanical model.*

**Proposition 1**  (Canonical Reformulation under Small Stiffness Perturbations)**.** *Let $\Omega \subset R^{2}$ be a bounded domain with sufficiently regular boundary, and let T>0. We assume the two hypotheses in Lemma 1 as well as $c,\delta ,K_{1}\in L^{\infty }\left(\Omega \times (0,T)\right)$, $p\in L^{\infty }\left(\Omega \right)$, λ∈C([0,T]). Moreover, assuming χ≥0, the condition $1+\chi I_{2}\left[w\right]\left(t\right)>0$ is automatically satisfied throughout the operating regime prior to pull-in (3). Accordingly, the normalized bending coefficient is represented as the sum of a unit reference value and a uniformly bounded perturbation κ(x,t):*

(4) $$K_{1}\left(x,t\right)=1+\kappa \left(x,t\right),$$

*with*

(5) $${∥\kappa ∥}_{L^{\infty }\left(\Omega \times \left(0,T\right)\right)}\leq \eta \ll 1.$$

*Substituting *(3)* and *(4)* into *(1)* yields*

(6) $$\begin{array}{c} \begin{array}{c} c\left(x,t\right)w_{t}\left(x,t\right)+w_{tt}\left(x,t\right)+\Delta^{2}w\left(x,t\right)=\\ =\left[\beta I_{1}\left[w\right]\left(t\right)+\gamma \right]\Delta w\left(x,t\right)+\frac{{\left(\lambda \left(t\right)\right)}^{2}p\left(x\right)}{{\left[1-w(x,t)\right]}^{2}{\left[1+\chi I_{2}\left[w\right]\left(t\right)\right]}^{2}}+\;\\ +\;\frac{{\left(\lambda \left(t\right)\right)}^{2}\delta \left(x,t\right){\left|\nabla w(x,t)\right|}^{2}}{{\left[1-w(x,t)\right]}^{2}}+R_{K}\left[w\right] \end{array} \end{array}$$

*with residual*

(7) $$R_{K}\left[w\right]\left(x,t\right)=-\kappa \left(x,t\right)\Delta^{2}w\left(x,t\right),$$

*which satisfies*

(8) $${∥R_{K}\left[w\right]\left(\cdot ,t\right)∥}_{L^{2}\left(\Omega \right)}\leq \eta {∥\Delta^{2}w\left(\cdot ,t\right)∥}_{L^{2}\left(\Omega \right)}.$$

*Therefore, when neglecting the O(η) residual under assumption η≪1, *(6)* becomes*

(9) $$\begin{array}{c} c\left(x,t\right)w_{t}\left(x,t\right)+w_{tt}\left(x,t\right)+\Delta^{2}w\left(x,t\right)=\left[\beta I_{1}\left[w\right]\left(t\right)+\gamma \right]\Delta w\;+\\ +\;\frac{{\left(\lambda \left(t\right)\right)}^{2}p\left(x\right)}{{\left[1-w(x,t)\right]}^{2}{\left[1+\chi I_{2}\left[w\right]\left(t\right)\right]}^{2}}+\frac{{\left(\lambda \left(t\right)\right)}^{2}\delta \left(x,t\right){\left|\nabla w(x,t)\right|}^{2}}{{\left[1-w(x,t)\right]}^{2}}. \end{array}$$

## 5. Lie Symmetry Classification

To apply Lie symmetry theory to (9), the nonlocal contributions are made explicit and the clamped boundary conditions are specified on each side of the rectangular domain. Since x=(x,y), the boundary conditions along the vertical edges x=±a/2 are given by

(10) $$w\left(\pm \frac{a}{2},y,t\right)=0,\;w_{x}\left(\pm \frac{a}{2},y,t\right)=0,\;-\frac{b}{2}\leq y\leq \frac{b}{2},$$

whereas on the horizontal edges y=±b/2, the clamped boundary conditions take the form

(11) $$w\left(x,\pm \frac{b}{2},t\right)=0,\;w_{y}\left(x,\pm \frac{b}{2},t\right)=0,\;-\frac{a}{2}\leq x\leq \frac{a}{2}.$$

### 5.1. Introduction of the Nonlocal Variables and Extended Differential Equation

To rewrite (9) as an extended system, we introduce the auxiliary nonlocal variables $A\left(t\right)=I_{1}\left[w\right]\left(t\right)$ and $B\left(t\right)=I_{2}\left[w\right]\left(t\right)$. The canonical governing equation can then be expressed in the compact form

(12) $$\begin{array}{c} \begin{array}{c} F\left[w,A,B\right]=w_{tt}+c\left(x,t\right)w_{t}+\Delta^{2}w-\left[\beta A(t)+\gamma \right]\Delta w\;+\\ -\;\frac{\lambda^{2}\left(t\right)p\left(x\right)}{{\left(1-w\right)}^{2}{\left[1+\chi B(t)\right]}^{2}}-\frac{\lambda^{2}\left(t\right)\delta \left(x,t\right){\left|\nabla w\right|}^{2}}{{\left(1-w\right)}^{2}}=\\ =w_{tt}+c\left(x,t\right)w_{t}+w_{xxxx}+2w_{xxyy}+w_{yyyy}-\left[\beta A(t)+\gamma \right]\left(w_{xx}+w_{yy}\right)\;-\\ -\;\frac{\lambda^{2}\left(t\right)p\left(x\right)}{{\left(1-w\right)}^{2}{\left[1+\chi B(t)\right]}^{2}}-\frac{\lambda^{2}\left(t\right)\delta \left(x,t\right)\left(w_{x}^{2}+w_{y}^{2}\right)}{{\left(1-w\right)}^{2}}=0. \end{array} \end{array}$$

The equation is supplemented by the integral constraints

(13) $$G_{1}\left[w,A\right]=A\left(t\right)-\int_{\Omega }\left(w_{x}^{2}+w_{y}^{2}\right)\;dx\;dy=0$$

and

(14) $$G_{2}\left[w,B\right]=B\left(t\right)-\int_{\Omega }\frac{1}{1-w}\;dx\;dy=0.$$

It is important to distinguish the exact reformulation step from the subsequent small-perturbation approximation. Starting from the original model (1) the decomposition (4) leads exactly to model in (6), provided that the residual term (7) is retained. Hence, no approximation is introduced in the passage (1)→(6). The approximation is introduced only in the subsequent passage (6)→(9), where the residual $R_{K}\left[w\right]$ is neglected under the assumption (5).

### 5.2. Infinitesimal Transformations

Let us consider a one-parameter local Lie group acting on the independent and dependent variables through the transformations listed in Table 3. The associated infinitesimal generator is

(15) $$X=\tau \frac{\partial }{\partial t}+\xi^{x}\frac{\partial }{\partial x}+\xi^{y}\frac{\partial }{\partial y}+\phi \frac{\partial }{\partial w}+\alpha \frac{\partial }{\partial A}+\zeta \frac{\partial }{\partial B}.$$

Here, ϕ is the infinitesimal component associated with *w*, whereas α and ζ are constrained by the variations of the integral functionals induced by transformations of *w*, the spatial coordinates, and when admissible, the computational domain. The corresponding infinitesimal variations of the nonlocal functionals, including the spatial Jacobian contribution and the first-prolongation terms, are reported in Table A2. Since *A* and *B* are global time-dependent variables, admissible transformations must also preserve $A_{x}=A_{y}=B_{x}=B_{y}=0$, further restricting α and ζ. The resulting compatibility conditions for the auxiliary infinitesimal components evaluated on the corresponding integral-constraint manifolds $G_{1}\left[w,A\right]=0$ and $G_{2}\left[w,B\right]=0$ are summarized in Table A5. The determining system follows by applying the fourth prolongation of *X* to the extended governing equation and auxiliary constraints, then splitting the resulting invariance conditions with respect to the independent derivatives of the displacement field. The resulting independent kernel and compatibility conditions are reported in Table A4, and the complete symbolic derivation was independently verified in MAPLE 2025.2.

### 5.3. Effect of the Electrostatic Singularity

Concerning the effect of the electrostatic singularity, the following proposition holds; its proof sketch is reported in Appendix B.

**Proposition 2.**  *Let $S=\left\{(t,x,w,A,B):w=1\right\}$ denote the fixed-gap contact manifold. Any admissible infinitesimal generator *(15)* that preserves S must satisfy ${\left.\phi (t,x,w,A,B)\right|}_{w=1}=0$. In particular, constant translations in the displacement variable, ϕ=c≠0, are inadmissible; likewise, a homogeneous displacement scaling ϕ=cw is incompatible with the fixed contact level w=1 unless c=0. The tangency condition ${\phi |}_{w=1}=0$ is necessary, but insufficient by itself to conclude that ϕ vanishes identically. The latter conclusion must be obtained from the determining equations of the complete prolonged electromechanical system together with the boundary and contact manifold invariance conditions.*

In particular, the determining equations associated with the prolonged governing equation imply $\phi_{w}=0$. Combining Proposition 2 with the determining equations of the complete prolonged electromechanical system yields that the admissible vertical component is independent of the displacement variable. Since the contact manifold invariance condition requires this component to vanish at w=1, it follows that ϕ≡0. Thus, the absence of a nontrivial continuous transformation of the displacement variable results from the joint action of the determining equations and the fixed-gap compatibility condition rather than from contact manifold invariance alone.

### 5.4. Time Translation in the Autonomous Case

If c=c(x), δ=δ(x), and $\lambda \left(t\right)=\lambda_{0}$, then the model is autonomous with respect to t and invariant under the transformation $t^{*}=t+\varepsilon$, for which the associated infinitesimal generator is $X_{t}=\frac{\partial }{\partial t}$. Conversely, if at least one of the coefficients c, δ, or λ depends explicitly on time, then the invariance conditions require τ=0 and the time translation symmetry is lost.

### 5.5. Consequences of the Rectangular Geometry

The centered rectangular domain is invariant under the discrete reflections $R_{x}:\left(x,y\right)\mapsto \left(-x,y\right)$ and $R_{y}:\left(x,y\right)\mapsto \left(x,-y\right)$ provided that the constitutive coefficients and initial conditions satisfy the parity conditions summarized in Table 4. Although not generated by nontrivial Lie infinitesimal generators, these discrete symmetries define the invariant functional space used for the reduced-order model. Accordingly,

(16) $$w\left(x,t\right)\approx \sum_{j=1}^{N}q_{j}\left(t\right)\varphi_{j}\left(x\right),$$

where the modal basis satisfies the clamped boundary conditions and preserves the reflection symmetries. Its rigorous construction is presented in Section 5.7.

### 5.6. Kernel Algebra of the Generic Class

For the generic electromechanical model, c(x,t), p(x), δ(x,t), and λ(t) are treated as arbitrary independent functions. As summarized in Table 5, the fixed rectangular geometry, clamped boundary conditions, heterogeneous coefficients, nonlocal couplings, and electrostatic singularity suppress nontrivial continuous spatial symmetries. Time translation is admitted only in the autonomous case, while spatial translations and rotations require homogeneous models on unbounded domains. Consequently, the admissible symmetries are those that are compatible with the complete physical boundary value problem, and they define the invariant functional space used for the reduced-order construction.

The results in Table 5 clarify the symmetry structure underlying the reduced-order construction. For the generic bounded MEMS problem, the fixed rectangular geometry, clamped boundary conditions, heterogeneous coefficients, nonlocal couplings, and fixed electrostatic singular manifold exclude nontrivial continuous spatial Lie symmetries, and hence spatial similarity reductions. However, this does not exclude discrete symmetries of the complete boundary value problem. In particular, the centered rectangular configuration may retain the reflections $R_{x}$ and $R_{y}$ introduced in Section 5.5 when the parity conditions in Table 4 are satisfied. Although not generated by the infinitesimal Lie algebra, these finite transformations define invariant parity subspaces suitable for Galerkin projection. Thus, continuous Lie classification identifies the admissible transformations and symmetry obstructions, while the surviving discrete reflections select the physically compatible modal subspace. Accordingly, the proposed construction is neither a classical Lie similarity reduction nor an orbit reduction of a continuous Lie group, instead combining continuous Lie classification with invariant subspace selection based on discrete symmetry to obtain the symmetry-preserving Galerkin reduction [57]. The role of the Lie analysis should be distinguished from that of the subsequent Galerkin projection. If the invariant parity class of the complete boundary value problem were known a priori, a conventional symmetry-preserving Galerkin method could directly restrict the approximation to the corresponding even–even modal subspace and would lead to the same projection step adopted here. The additional contribution of the Lie analysis is not a different Galerkin projection operator but a systematic classification performed prior to reduction. In particular, it determines which continuous transformations are compatible with the governing equation, nonlocal constraints, electrostatic contact manifold, constitutive coefficients, geometry, and boundary conditions as well as which candidate symmetries are obstructed by these ingredients. For the present bounded MEMS problem, this analysis proves that nontrivial continuous spatial symmetries cannot be used for similarity reduction and that time translation survives only in the autonomous subclass. The subsequent analysis of the remaining discrete transformations then establishes the precise parity conditions under which the reflection-invariant subspaces are dynamically admissible. Thus, whereas a conventional symmetry-preserving Galerkin reduction starts from an assumed symmetry-compatible basis, the present Lie-classification/Galerkin workflow provides an a priori structural test of whether such a restriction is justified for the complete electromechanical problem and indicates when it must be modified due to changing coefficients, forcing, geometry, or boundary conditions.

### 5.7. Construction of a Symmetry-Preserving Modal Basis

Let ${\left\{\phi_{j}\right\}}_{j=1}^{\infty }$ be a complete family of modal functions compatible with the geometry, clamped boundary conditions, and discrete reflection symmetries. A natural choice is provided by the eigenfunctions of the clamped biharmonic operator, defined by the spectral problem $\Delta^{2}\phi_{j}=\mu_{j}\phi_{j}$ in Ω, with  $\phi_{j}=0$ and $\frac{\partial \phi_{j}}{\partial n}=0$ on ∂Ω. For the even–even symmetry class, the additional conditions $\phi_{j}\left(-x\right)=\phi_{j}\left(x\right)$ and $\phi_{j}\left(x,-y\right)=\phi_{j}\left(x\right)$ are imposed. The clamped biharmonic operator is self-adjoint and positive definite; hence, its eigenfunctions can be chosen as an orthonormal basis in $L^{2}\left(\Omega \right)$ [57].

### 5.8. Modal Approximation and Reduced Nonlinear Functionals

The reduced subspace is defined as $V_{N}=\mathrm{span}\left\{\phi_{1},\ldots ,\phi_{N}\right\}$. The modal approximation of the displacement is

(17) $$w_{N}\left(x,t\right)=\sum_{j=1}^{N}q_{j}\left(t\right)\phi_{j}\left(x\right),$$

where $q_{j}\left(t\right)$ are the generalized coordinates collected in q(t). The compact matrix notation for the modal basis, generalized coordinates, modal approximation, and associated temporal and spatial derivatives are summarized in Table A9. Since each $\phi_{j}$ belongs to $H_{0}^{2}\left(\Omega \right)$, the modal approximation identically satisfies $w_{N}=0$ on ∂Ω and $\partial w_{N}/\partial n=0$ on ∂Ω. Moreover, $w_{N}\left(-x,y,t\right)=w_{N}\left(x,y,t\right)$ and $w_{N}\left(x,-y,t\right)=w_{N}\left(x,y,t\right)$. The admissible and uniformly admissible sets of reduced coordinates are defined as

(18) $$Q_{N}=\left\{q\in R^{N}:1-\Phi^{T}\left(x\right)q>0\;\mathrm{for}\;a.e.\;x\in \Omega \right\},$$

and, for a prescribed safety margin $\varepsilon_{g}>0$,

(19) $$Q_{N,\varepsilon_{g}}=\left\{q\in R^{N}:1-\Phi^{T}\left(x\right)q\geq \varepsilon_{g}\;\mathrm{for}\;a.e.\;x\in \Omega \right\}.$$

These respectively conditions enforce positivity of the electrode gap and a uniform safety margin from the contact configuration. Substituting (17) into the stretching functional immediately yields the reduced functional $I_{1,N}\left(q\right)=\int_{\Omega }{\left|\nabla w_{N}\right|}^{2}\;d\Omega$. Similarly, $I_{2,N}\left(q\right)\;=\;\int_{\Omega }\frac{1}{1-\Phi^{T}\left(x\right)q}\;d\Omega$. The reduced stretching functional together with its compact matrix representation, gradient, and Hessian is summarized in Table A7. First- and second-order derivatives are reported in Table A8.

### 5.9. Galerkin Projection and Mechanical Contributions

The Galerkin projection is obtained by evaluating the residual of the canonical continuum model on the reduced modal approximation and enforcing its orthogonality to each basis function of the symmetry-preserving modal space. The residual includes the inertial, damping, bending, pre-stress, geometric stretching, electrostatic, and $E_{\mathrm{ff}}$ contributions, as detailed in Table A10. Projection of the mechanical terms yields the reduced mass, damping, bending, and stretching matrices M, C(t), $K^{\left(b\right)}$, and G, respectively, as reported in Table A11. Accordingly, the overall linear mechanical stiffness matrix is $K=K^{\left(b\right)}+\gamma G$, whereas the nonlinear mechanical contribution assumes the compact form

(20) $$f_{m}\left(q\right)=Kq+\beta \left(q^{T}Gq\right)Gq.$$

### 5.10. Reduction of the Electrostatic and $E_{\mathrm{ff}}$ Contributions

The Galerkin projection of the electrostatic pressure yields the reduced electrostatic load vector

(21) $$P\left(q\right)=\int_{\Omega }\frac{p(x)\Phi (x)}{{\left[1-\Phi^{T}\left(x\right)q\right]}^{2}}\;d\Omega ,$$

and the capacitive feedback is represented through $H\left(q\right)=1+\chi I_{2,N}\left(q\right)$ so that $f_{\mathrm{es}}\left(q,t\right)\;=\;\lambda^{2}\left(t\right)P\left(q\right)/H^{2}\left(q\right)$. Similarly, the Galerkin projection of the $E_{\mathrm{ff}}$ contribution is written as

(22) $$D\left(q,t\right)=\int_{\Omega }\frac{\delta \left(x,t\right)\Phi \left(x\right){\left|\nabla \;\left(\Phi^{T}\left(x\right)q\right)\right|}^{2}}{{\left[1-\Phi^{T}\left(x\right)q\right]}^{2}}\;d\Omega ,$$

yielding the generalized $E_{\mathrm{ff}}$ force $f_{\mathrm{ff}}\left(q,t\right)=\lambda^{2}\left(t\right)D\left(q,t\right)$. For completeness, the analytical expressions of the electrostatic tangent matrix together with the Jacobian matrices associated with P(q) and D(q,t) are reported in Table A3.

### 5.11. Lie-ROM System with *N* DoF

By collecting all contributions, the reduced-order system is obtained as follows:

(23) $$\begin{array}{c} M\ddot{q}+C\left(t\right)\dot{q}+Kq+\beta \left(q^{T}Gq\right)Gq= \end{array}$$

(24) $$\begin{array}{c} =\frac{\lambda^{2}\left(t\right)}{{\left[1+\chi I_{2,N}\left(q\right)\right]}^{2}}P\left(q\right)+\lambda^{2}\left(t\right)D\left(q,t\right)+r_{\eta ,N}\left(q,t\right) \end{array}$$

where $r_{\eta ,N}$ denotes the projected residual associated with the small flexural stiffness perturbation, and is neglected for η≪1. The mechanical energy structure of the reduced-order system, including the associated conservation law and general energy balance, is summarized in Table A6.

### 5.12. Residual Perturbation and Reduced Initial Conditions

Writing the reduced dynamics in first-order form, the resulting vector field is locally Lipschitz continuous with respect to the state variables under the assumed regularity conditions and for $q\in Q_{N,\varepsilon_{g}}$. Hence, the Picard–Lindelöf theorem [58] guarantees existence and uniqueness of a local solution, which can be continued as long as the trajectory remains bounded and within the admissible set. Loss of continuability may occur only when ${\mathrm{ess inf}}_{(x)\in \Omega }\left[1-\Phi^{T}\left(x\right)q\left(t\right)\right]⟶0^{+}$, corresponding to the onset of the electrostatic singularity. The initial coordinates are obtained by means of the $\left(L^{2}\right)$-projection of the continuous initial data. Specifically, we define

(25) $${\left(b_{0}\right)}_{i}=\int_{\Omega }w_{0}\left(x\right)\phi_{i}\left(x\right)\;d\Omega ,\;{\left(b_{1}\right)}_{i}=\int_{\Omega }v_{0}\left(x\right)\phi_{i}\left(x\right)\;d\Omega$$

so that $q\left(0\right)=M^{-1}b_{0}$ and $\dot{q}\left(0\right)=M^{-1}b_{1}$.

### 5.13. Static Equilibria

For time-independent coefficients and constant actuation, $\lambda \left(t\right)=\lambda_{0}$, static equilibria satisfy $\dot{q}=0$ and $\ddot{q}=0$ with static residual    

(26) $$R_{s}\left(q,\lambda_{0}\right)=Kq+\beta \left(q^{T}Gq\right)Gq-\frac{\lambda_{0}^{2}}{{\left[1+\chi I_{2,N}\left(q\right)\right]}^{2}}P\left(q\right)-\lambda_{0}^{2}D\left(q\right).$$

Therefore, a static equilibrium $q_{s}$ is characterized by $R_{s}\left(q_{s},\lambda_{0}\right)=0$ and the mechanical tangent stiffness matrix is given by

(27) $$K_{m,T}\left(q\right)=K+\beta \left(q^{T}Gq\right)G+2\beta \left(Gq\right){\left(Gq\right)}^{T}.$$

### 5.14. Static Pull-In Condition and Linearization About a Biased Equilibrium

A limit point of the equilibrium branch is characterized by the coupled conditions $R_{s}\left(q_{\mathrm{PI}},\lambda_{\mathrm{PI}}\right)=0$ and $\det\left[K_{T}\left(q_{\mathrm{PI}},\lambda_{\mathrm{PI}}\right)\right]=0$, or equivalently by the existence of a nonzero vector $v_{\mathrm{PI}}$ satisfying $K_{T}\left(q_{\mathrm{PI}},\lambda_{\mathrm{PI}}\right)v_{\mathrm{PI}}=0$, $v_{\mathrm{PI}}^{T}v_{\mathrm{PI}}=1$. Let $q_{s}$ be a stable static equilibrium. Introducing the perturbation $q\left(t\right)=q_{s}+\tilde{q}\left(t\right)$, where $\tilde{q}\left(t\right)$ denotes a small perturbation about the static equilibrium $q_{s}$, the reduced dynamics linearizes to $M\ddot{\tilde{q}}+C\dot{\tilde{q}}+K_{T}\left(q_{s},\lambda_{0}\right)\tilde{q}=0$. Moreover, $K_{T}\left(q_{s},\lambda_{0}\right)u_{r}=\omega_{r}^{2}Mu_{r}$, where $\omega_{r}$ and $u_{r}$ denote the *r*th natural angular frequency and corresponding modal eigenvector, respectively. Linear static stability requires $\omega_{r}^{2}>0$ for all modes, while $\min_{r}\omega_{r}^{2}\to 0^{+}$ at pull-in, indicating the loss of stiffness associated with electrostatic instability.

## 6. Physics-Informed One-Degree-of-Freedom Lie-ROM

The general *N*-DoF Lie-ROM preserves the admissible symmetries and dominant electromechanical mechanisms of the continuum model. For computational efficiency, it is specialized here to the monomodal case (N=1), using a single mode consistent with the clamped boundary conditions and reflection symmetries. Thus, the matrix operators reduce to scalar coefficients while geometric stretching, capacitive feedback, dielectric inhomogeneity, and fringing field effects remain embedded in the reduced nonlinear functionals. The resulting one-DoF Lie-ROM is the symmetry-consistent and physically interpretable model adopted for our numerical investigations.

### 6.1. Monomodal Approximation and Reduced Nonlinear Functionals

Under the monomodal approximation, the distributed electromechanical contributions reduce to nonlinear scalar functionals of the generalized coordinate q(t), while retaining geometric stretching, capacitive feedback, dielectric inhomogeneity, and fringing field effects through the symmetry-preserving mode. The modal coefficients, reduced functionals, generalized forces, and analytical derivatives required for model assembly, tangent stiffness evaluation, stability analysis, and pull-in prediction are summarized in Table 6 in order to separate the analytical formulation from its computational implementation.

### 6.2. Governing Equation and Linearization

The reduced equation of motion becomes

(28) $$m\;\ddot{q}+c\;\dot{q}+k\;q+\beta g^{2}q^{3}=\frac{\lambda^{2}\left(t\right)}{H^{2}\left(q\right)}\;P\left(q\right)+\lambda^{2}\left(t\right)\;D\left(q,t\right)+r_{\eta },$$

where *m*, *c*, *k*, and *g* denote the reduced inertial, damping, bending, and geometric coefficients, respectively, while H(q)=1+χB(q) represents the reduced capacitive feedback factor associated with the deformation-dependent capacitance. Their explicit analytical expressions are summarized in Table 6. Since the monomodal model is a specialization of the general *N*-DoF Lie-ROM, the required first- and second-order derivatives are inherited directly from the general formulation. In the monomodal case, the tangent operator reduces to the scalar derivative of the residual with respect to *q* and incorporates the reduced capacitive functional, dielectric projection functional, $E_{\mathrm{ff}}$ contribution, and associated auxiliary integrals. These analytical quantities provide the consistent linearization of the reduced model.

### 6.3. Static Equilibrium and Pull-In Analysis

The static response of (28) is obtained for constant voltage actuation, vanishing generalized velocity and acceleration, and under the small stiffness perturbation approximation $r_{\eta }=0$ adopted in the reduced formulation. Under these conditions, the nonlinear dynamic problem reduces to the equilibrium equation R(q,λ)=0, where the nonlinear residual is given by $R\left(q,\lambda \right)=kq+k_{3}q^{3}-\lambda^{2}\left[\frac{P(q)}{{\left(1+\chi B(q)\right)}^{2}}+D\left(q\right)\right]$, where $k_{3}=\beta g^{2}$ denotes the reduced cubic stretching coefficient. The corresponding tangent operator is obtained from the Jacobian of the residual, $K_{T}\left(q,\lambda \right)=\frac{\partial R(q,\lambda )}{\partial q}$. The analytical tangent stiffness follows directly from the derivative of the reduced residual. The loss of static stability is associated with the classical pull-in instability. In the reduced framework, the pull-in point is identified as the limit point of the equilibrium branch and is characterized by the simultaneous fulfillment of the equilibrium and singularity conditions $R(q_{\mathrm{PI}},\lambda_{\mathrm{PI}})=0$, $K_{T}\left(q_{\mathrm{PI}},\lambda_{\mathrm{PI}}\right)=0$. These two coupled conditions provide the mathematical definition of the static pull-in point, whereas their numerical localization and refinement along the equilibrium branch are performed through the continuation procedure described in Section 7.5.

## 7. Numerical Implementation and Comparative Modeling Framework

### 7.1. Common Physical Configuration and Parameter Set

For a consistent comparison, all models share the same physical configuration, material and geometric properties, loading conditions, constitutive coefficients, boundary and initial conditions, voltage history, and numerical convergence criteria unless otherwise specified. All simulations were performed in MATLAB Release 2026a within the same computational environment. Hardware specifications are reported in Table 7, while numerical tolerances and convergence criteria are summarized in Table 8.

### 7.2. High-Fidelity Continuum Model

Model (9) is adopted as the high-fidelity full-order model (FOM) and provides the reference solution for all numerical comparisons. Its modular implementation separately evaluates the distributed mechanical operators, electrostatic loading, geometric stretching, capacitive feedback, dielectric distribution, and $E_{\mathrm{ff}}$ correction before assembling the nonlinear electromechanical system. Static equilibria are computed by a Newton–Raphson procedure using the analytical tangent operator, while pull-in is determined by incremental voltage continuation until loss of equilibrium stability. Transient responses are obtained by integrating the nonlinear dynamic system and updating the distributed contributions and nonlocal functionals at each time step. The software comprises independent modules for parameter initialization, operator evaluation, model assembly, static and dynamic analyses, and postprocessing.

### 7.3. Lie-ROM Offline–Online Implementation

The model in (28) was implemented within the computational framework of Section 7.2 using an offline–online decomposition. The symmetry-preserving modal basis was obtained from the discrete clamped biharmonic eigenvalue problem associated with the spatial discretization of Section 7.5. The first six eigenpairs of the sparse biharmonic operator were computed with MATLAB eigs using the smallest-magnitude target, and the lowest mode satisfying the prescribed even–even reflection symmetries was retained. The resulting mode ϕ(x) was normalized according to $\int_{\Omega }\phi^{2}\left(x\right)\;dx=1$ using the FOM spatial quadrature, and its parity was verified numerically with respect to both coordinate reflections. In the offline stage, the normalized mode and the reduced quantities reported in Table 6 were evaluated once and stored using the same grid and composite quadrature as the continuum model. Online computations update only the generalized coordinate, while the stretching, capacitive feedback, electrostatic, and $E_{\mathrm{ff}}$ contributions are evaluated through their closed-form reduced expressions. The analytical derivatives in Table 6 provide the tangent operator without numerical differentiation, thereby improving the robustness of the Newton iterations. Static equilibria, pull-in conditions, and transient responses were computed using the same numerical procedures and convergence criteria as model (9). Owing to the single-DoF formulation, the online cost is dominated by nonlinear scalar evaluations rather than by the solution of the original fourth-order integro-partial differential equation.

### 7.4. Classical Lumped-Parameter Model

For comparison, a classical single-DoF lumped-parameter model is constructed for the same reference microplate adopted for the FOM and the proposed Lie-ROM, following the nonlinear mass–spring–damper formulation with parallel-plate electrostatic actuation reported in [55]. The equivalent mechanical coefficients are derived from the same geometry, material properties, pre-stress, damping, and fundamental mode used in the monomodal formulation, without fitting to either the FOM or Lie-ROM response. To make the lumped coordinate directly representative of the normalized central displacement, the fundamental mode is normalized as $\psi \left(x\right)=\frac{\phi (x)}{\phi (0)}$, ψ(0)=1. This normalization is introduced only for the lumped benchmark, and does not alter the modal normalization adopted in the Lie-ROM. The equivalent lumped mass and damping coefficients are

(29) $$m_{L}=\int_{\Omega }\psi^{2}\left(x\right)\;d\Omega ,\;c_{L}\left(t\right)=\int_{\Omega }c\left(x,t\right)\psi^{2}\left(x\right)\;d\Omega .$$

For spatially uniform damping, $c\left(x,t\right)=c_{0}$, $c_{L}=c_{0}m_{L}$. The bending and pre-stress contributions are defined through

(30) $$k_{L}^{\left(b\right)}=\int_{\Omega }{\left[\Delta \psi (x)\right]}^{2}\;d\Omega ,\;g=\int_{\Omega }{\left|\nabla \psi (x)\right|}^{2}\;d\Omega .$$

Thus, $k_{L}=k_{L}^{\left(b\right)}+\gamma g$ and  $k_{3,L}=\beta g^{2}$, with all the lumped mechanical coefficients derived from the same modal and physical properties as the Lie-ROM. The resulting classical lumped model is

(31) $$m_{L}{\left(w_{L}\right)}_{tt}+c_{L}{\left(w_{L}\right)}_{t}+k_{L}w_{L}+k_{3,L}w_{L}^{3}=\frac{{\left(\lambda \left(t\right)\right)}^{2}}{{\left(1-w_{L}\right)}^{2}},\;0\leq w_{L}<1,$$

where, because ψ(0)=1, $w_{L}\left(t\right)$ directly represents the normalized central displacement. Equation (31) retains the conventional parallel-plate inverse-square electrostatic force. Unlike the Lie-ROM (28), it does not explicitly include the distributed dielectric weighting p(x), the nonlocal capacitive feedback functional, or the distributed $E_{\mathrm{ff}}$ contribution. Thus, the two one-DoF models refer to the same physical device and deformation pattern but preserve different levels of distributed electromechanical information. For constant actuation, $\lambda \left(t\right)=\lambda_{0}$, with the static equilibrium governed by

(32) $$R_{L}\left(w_{L},\lambda_{0}\right)=k_{L}w_{L}+k_{3,L}w_{L}^{3}-\frac{\lambda_{0}^{2}}{{\left(1-w_{L}\right)}^{2}}=0.$$

For constant actuation, the static equilibrium is governed by (32) and the corresponding pull-in point is obtained from $R_{L}=0$ together with $K_{L,T}=0$. No equivalent coefficient is calibrated against the FOM or Lie-ROM; therefore, differences in static response, pull-in voltage, and transient dynamics arise from the modeling assumptions rather than from independently fitted parameters. For the numerical comparisons, $w_{L}\left(t\right)$ is compared with the normalized central displacements $w_{c,\mathrm{FOM}}\left(t\right)=w\left(0,t\right)$ and $w_{c,\mathrm{Lie}}\left(t\right)=q\left(t\right)\phi \left(0\right)$. The latter retains the original Lie-ROM modal normalization, whereas ψ is used only to define the lumped benchmark. The three models are subjected to the same voltage histories, initial conditions, damping level, and numerical convergence criteria. Thus, the FOM provides the distributed reference solution, the Lie-ROM its physics-informed symmetry-preserving one-DoF reduction, and (31) a conventional one-DoF benchmark for quantifying the benefit of retaining the distributed electromechanical structure.

### 7.5. Solvers, Tolerances, Continuation, and Admissibility Criteria

The FOM was spatially discretized on a uniform structured rectangular grid using second-order centered finite differences. The final simulations employed a 121×81 grid, corresponding to $N_{\mathrm{FOM}}=9801$ displacement values. Boundary values were included in this count for a uniform compression ratio measure, although the clamped values were not treated as independent unknowns. Spatial derivatives were implemented through MATLAB sparse matrix operators, with the biharmonic term assembled from the discrete Laplacian and the clamped conditions w=0 and $\partial_{n}w=0$ enforced through the corresponding boundary finite-difference relations. The spatial integrals associated with geometric stretching, capacitive feedback, dielectric effects, and electrostatic loading were evaluated by composite trapezoidal quadrature using MATLAB trapz along both spatial directions. Sparse matrices were assembled with sparse and spdiags, and the linear systems arising within the nonlinear iterations were solved using MATLAB’s built-in sparse linear algebra routines. Static equilibria were computed by Newton–Raphson iterations using the analytical tangent operator and the MATLAB backslash operator (\). The equilibrium branch was traced by incremental voltage continuation, using the previously converged solution as the initial guess. The initial increment was $\Delta V_{0}=1\;V$; it was retained when convergence occurred within five Newton iterations and otherwise successively halved. Near pull-in, identified when the smallest tangent-stiffness eigenvalue dropped below 5% of its zero-voltage value, the increment was reduced to ΔV≤0.1V, with $\Delta V_{\min}=10^{-3}\;V$. The critical voltage was refined until successive estimates differed by less than $10^{-3}\;V$. Pull-in was identified from equilibrium together with loss of tangent stiffness. For the FOM pull-in was evaluated through the vanishing of the smallest tangent stiffness eigenvalue using MATLAB eigs, while for the monomodal Lie-ROM it was evaluated through $K_{T}=0$. Transient responses were integrated with MATLAB ode45 using the adaptive explicit Dormand–Prince Runge–Kutta scheme with $\mathrm{RelTol}=10^{-8}$ and $\mathrm{AbsTol}=10^{-10}$. These tolerances were selected through preliminary refinement tests to ensure negligible changes in displacement histories, pull-in voltage, and energy response. Throughout the static and transient analyses, the admissibility condition $1-\Phi^{T}\left(x\right)q>0$ was monitored over the complete computational domain; branches entering nonphysical configurations were terminated. CPU times were measured for all models on the same hardware and MATLAB environment, and were averaged over 20 independent runs. Model initialization, offline preprocessing, and graphical postprocessing were excluded from the online CPU measurements, whereas the Lie-ROM offline construction time was measured separately. The main numerical settings are summarized in Table 8.

### 7.6. Error Metrics and Computational Indicators

The numerical accuracy of the model in (28) relative to the FOM is assessed by comparison with the continuum model, and where appropriate with the model in (31) using the quantitative performance indicators summarized in Table 9. Static accuracy is evaluated through the relative error of the transverse displacement at the plate center (computed only for $|w_{c,\mathrm{FOM}}|>0$) and the critical pull-in voltage, whereas transient accuracy is measured from the complete central displacement histories and the corresponding normalized error norm. Computational efficiency is quantified through CPU speed-up and reduction in the number of degrees of freedom. For the model in (28), physical consistency is further assessed by the energy conservation and symmetry preservation indicators. Here, FOM denotes the model in (9), whereas “model” refers either to the model in (28) or the one in (31), depending on the comparison under consideration. The symmetry preservation indicator is evaluated only for the FOM and the model in (28), since the lumped model in (31) does not reconstruct a distributed displacement field.

## 8. Numerical Results and Discussion

### 8.1. Spatial Convergence

**Figure 3:**
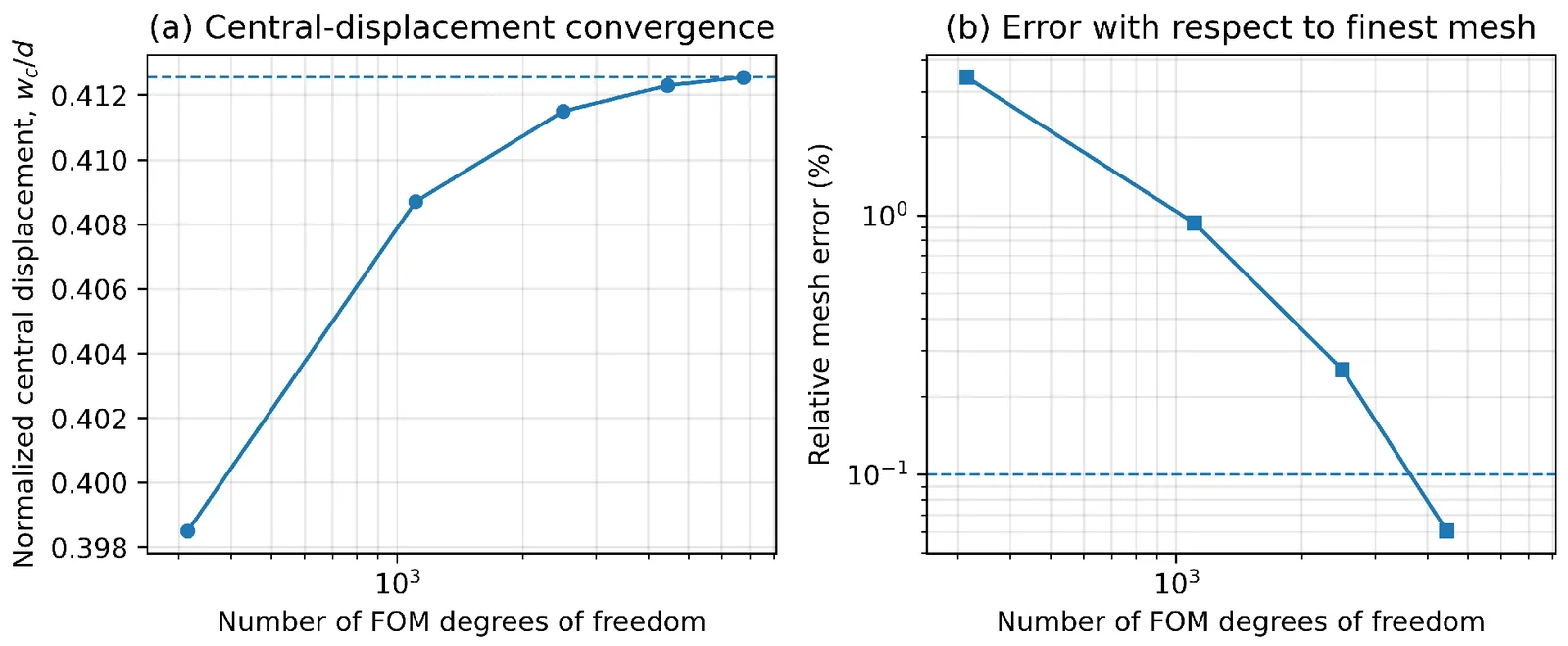
Spatial convergence of the continuum model at $V=0.8\bar{V_{\mathrm{PI}}^{\mathrm{FOM}}}$: (**a**) normalized central displacement and (**b**) relative error. The dashed line indicates the 0.1% convergence threshold.

Before comparing the reduced-order models with the continuum formulation, the spatial discretization of the high-fidelity reference solution is verified through a mesh convergence study at $V=0.8V_{\mathrm{PI}}^{\mathrm{FOM}}$, a representative condition before pull-in that is characterized by pronounced nonlinear electromechanical coupling. As shown in the left panel of Figure 3, the normalized central displacement rapidly converges to a mesh-independent value with increasing spatial resolution. Correspondingly, the relative error in the right panel of Figure 3 decreases monotonically and falls below the prescribed 0.1% threshold for the reference mesh adopted in the subsequent simulations. The resulting FOM solution can be regarded as spatially converged, ensuring that subsequent discrepancies with the Lie-ROM and the model in (31) primarily reflect their intrinsic approximation properties rather than spatial discretization errors.

### 8.2. Temporal Convergence

Temporal convergence of the FOM was assessed for a voltage step $V_{0}=0.60V_{\mathrm{PI}}^{\mathrm{FOM}}$ by progressively reducing the maximum integration step. As summarized in Table 10, the peak displacement error decreases from 0.745% at $\Delta t_{\max}=2.00\;\mu s$ to 0.039% at $\Delta t_{\max}=0.50\;\mu s$, using the solution obtained with $\Delta t_{\max}=0.25\;\mu s$ as the reference. Accordingly, $\Delta t_{\max}\;=\;0.50\;\mu s$ was adopted for the subsequent transient simulations and computational-efficiency benchmarks. This setting provides temporal convergence within 0.05% while reducing the computational cost relative to the finer $\Delta t_{\max}=0.25$ μs reference solution. Therefore, the FOM CPU time used in the subsequent performance comparisons corresponds to $\Delta t_{\max}=0.50\;\mu s$.

### 8.3. Static Response and Modal Convergence

**Figure 4:**
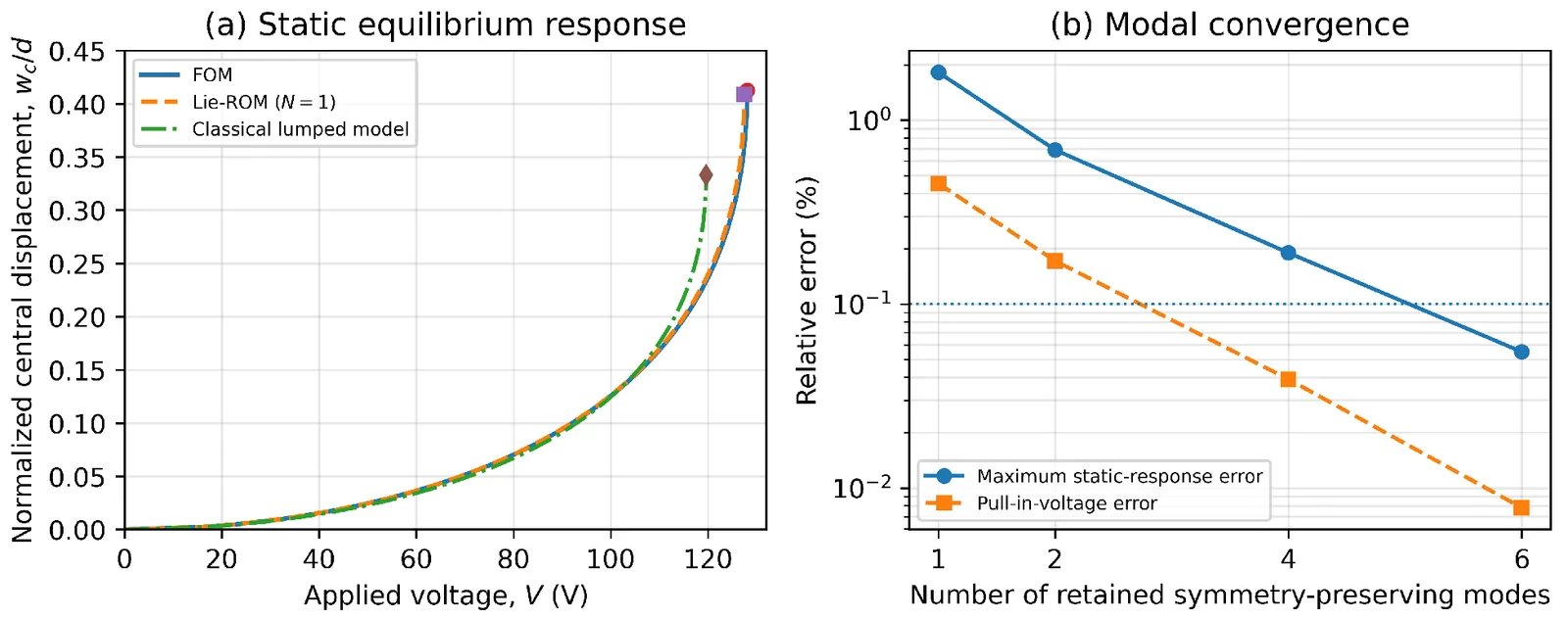
Static response and modal convergence of the proposed Lie-ROM. (**a**) Normalized central displacement versus applied voltage for the FOM, monomodal Lie-ROM, and classical lumped model. (**b**) Maximum static response error and pull-in voltage error versus the number *N* of retained symmetry-preserving modes. The multimodal Lie-ROMs (N>1) are considered only for the modal convergence assessment, with the monomodal formulation (N=1) adopted for the subsequent numerical benchmarks.

The static performance of the proposed Lie-ROM is assessed against the high-fidelity continuum model over the complete operating range prior to pull-in. As shown in the left panel of Figure 4, the monomodal Lie-ROM closely reproduces the nonlinear central displacement response of the FOM up to the onset of pull-in. The modal convergence analysis in the right panel of Figure 4 shows a rapid decrease in both static response and pull-in voltage errors as the reduced basis is enriched, indicating that the dominant nonlinear electromechanical behavior is already captured by the fundamental symmetry-preserving mode. The corresponding pull-in predictions are summarized in Table 11. The monomodal Lie-ROM predicts the pull-in voltage with a relative error of only 0.37% while reducing the model from 9801 FOM displacement values to a single generalized coordinate. In contrast, the classical lumped-parameter model, despite having the same reduced dimension, yields a pull-in error of 6.04%. These results show that the symmetry-informed reduced space provides an effective single-DoF representation while retaining the dominant distributed nonlinear electromechanical mechanisms of the continuum model.

**Figure 5:**
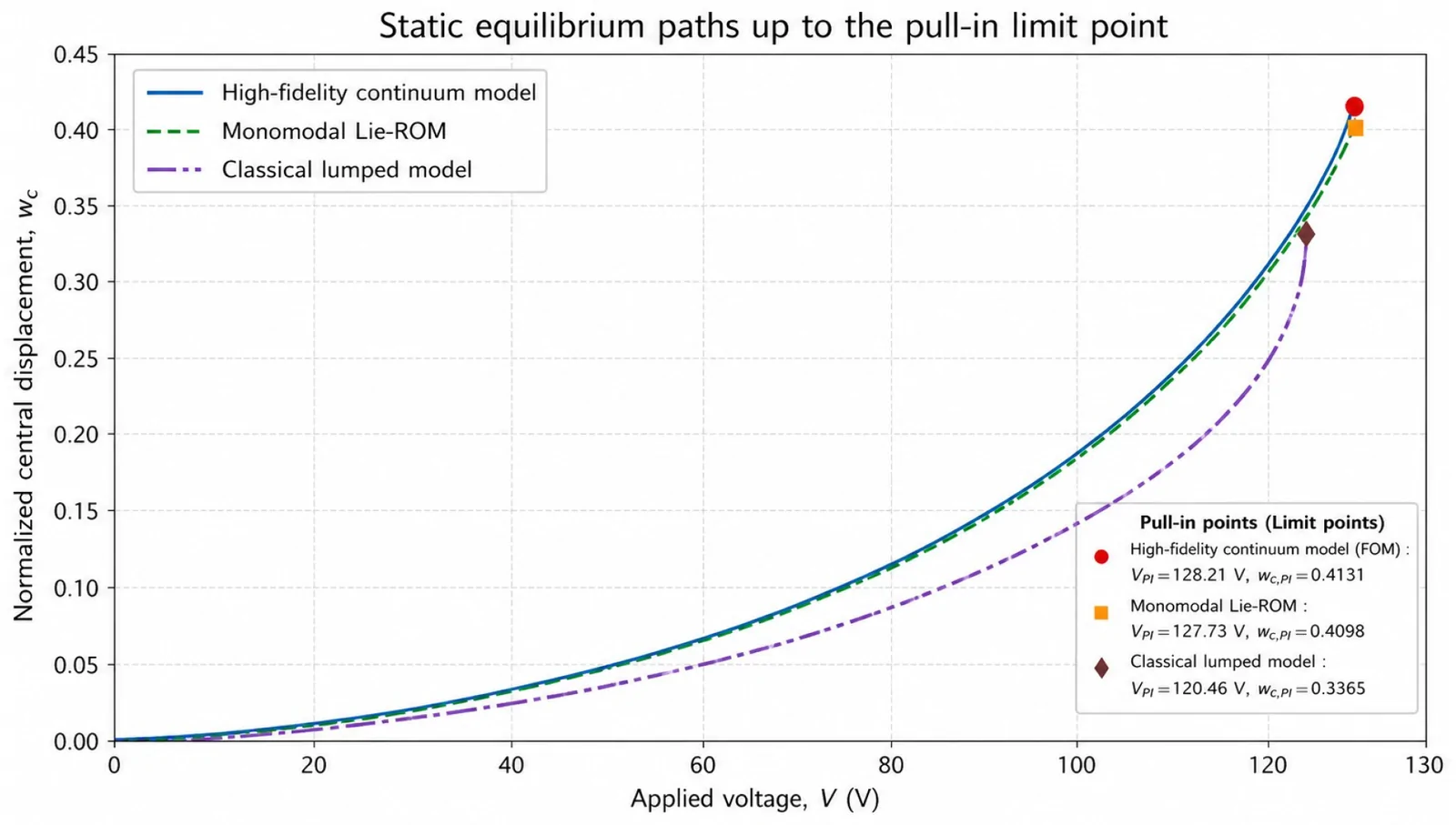
Static equilibrium paths of the FOM, monomodal Lie-ROM, and classical lumped-parameter model. Markers indicate the pull-in points.

As shown in Table 11, the proposed Lie-ROM predicts a pull-in voltage of 127.73V, compared with 128.21V for the FOM. The corresponding signed error is −0.37%, indicating a slight underprediction of the pull-in threshold, while the absolute discrepancy remains below 0.4%. By comparison, the lumped model exhibits a substantially larger signed error of −6.04%. Therefore, the negative Lie-ROM error reveals a small conservative tendency of the monomodal approximation for the nominal configuration. The corresponding static equilibrium branches predicted by the FOM, monomodal Lie-ROM, and classical lumped-parameter model are compared in Figure 5, where the respective pull-in points are also highlighted.

### 8.4. Dynamic Response Under Step and Harmonic Actuation

**Figure 6:**
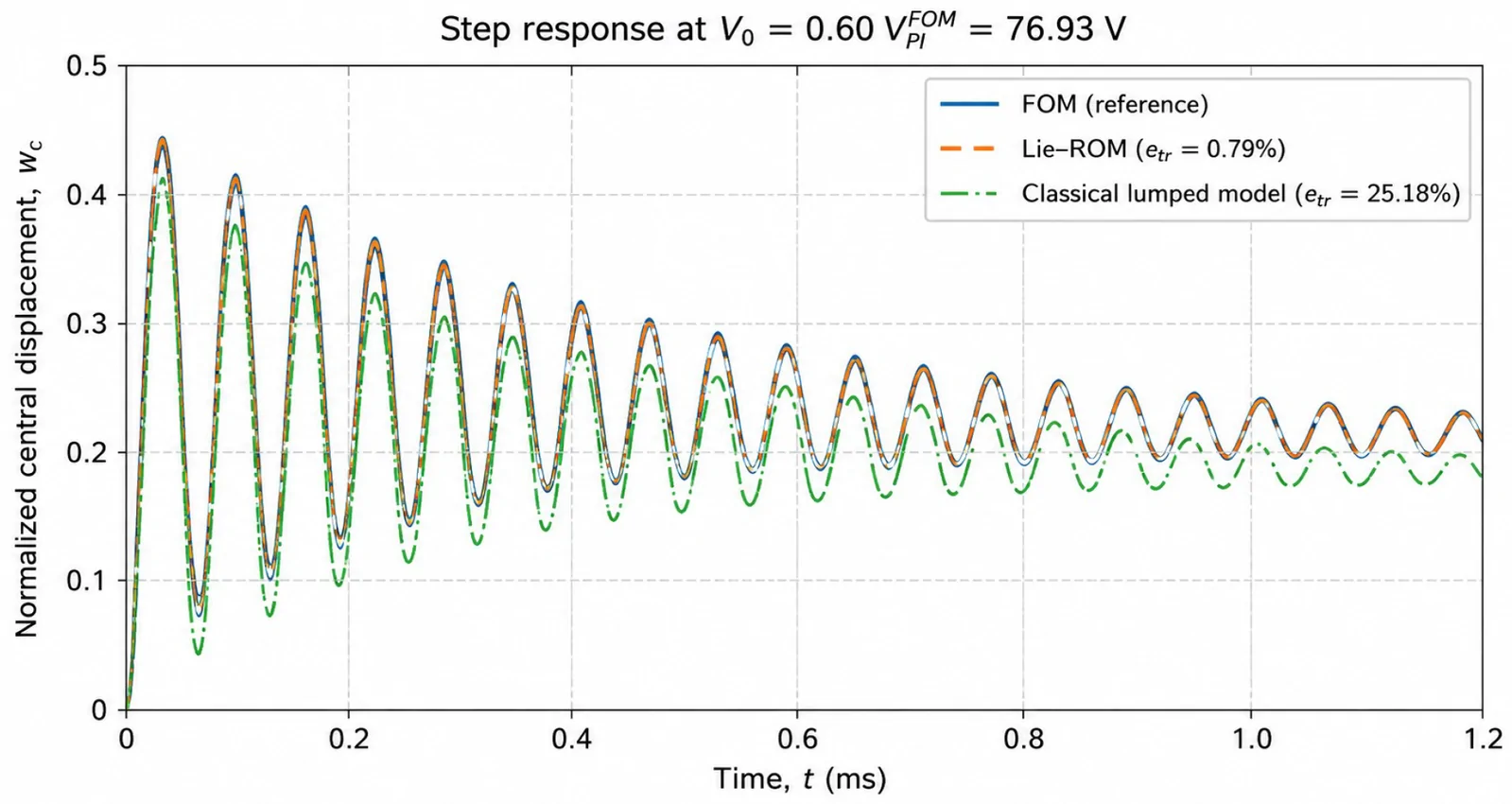
Comparison of the transient central displacement response ($w_{c}/d$) predicted by the FOM, proposed Lie-ROM, and classical lumped model under step actuation ($V_{0}=0.60V_{\mathrm{PI}}^{\mathrm{FOM}}$, Q=50).

The dynamic performance of the model (28) was first assessed under step actuation from the initially undeformed configuration, with $V_{0}=0.60V_{\mathrm{PI}}^{\mathrm{FOM}}=76.93\;V$. As shown in Figure 6, (28) remains almost indistinguishable from model (9) throughout the simulation, yielding a normalized transient error of $e_{\mathrm{tr}}=0.79\%$. In contrast, model (31) underestimates both the transient peak and steady displacement. It also exhibits a noticeable phase discrepancy, resulting in $e_{\mathrm{tr}}=25.18\%$.

**Figure 7:**
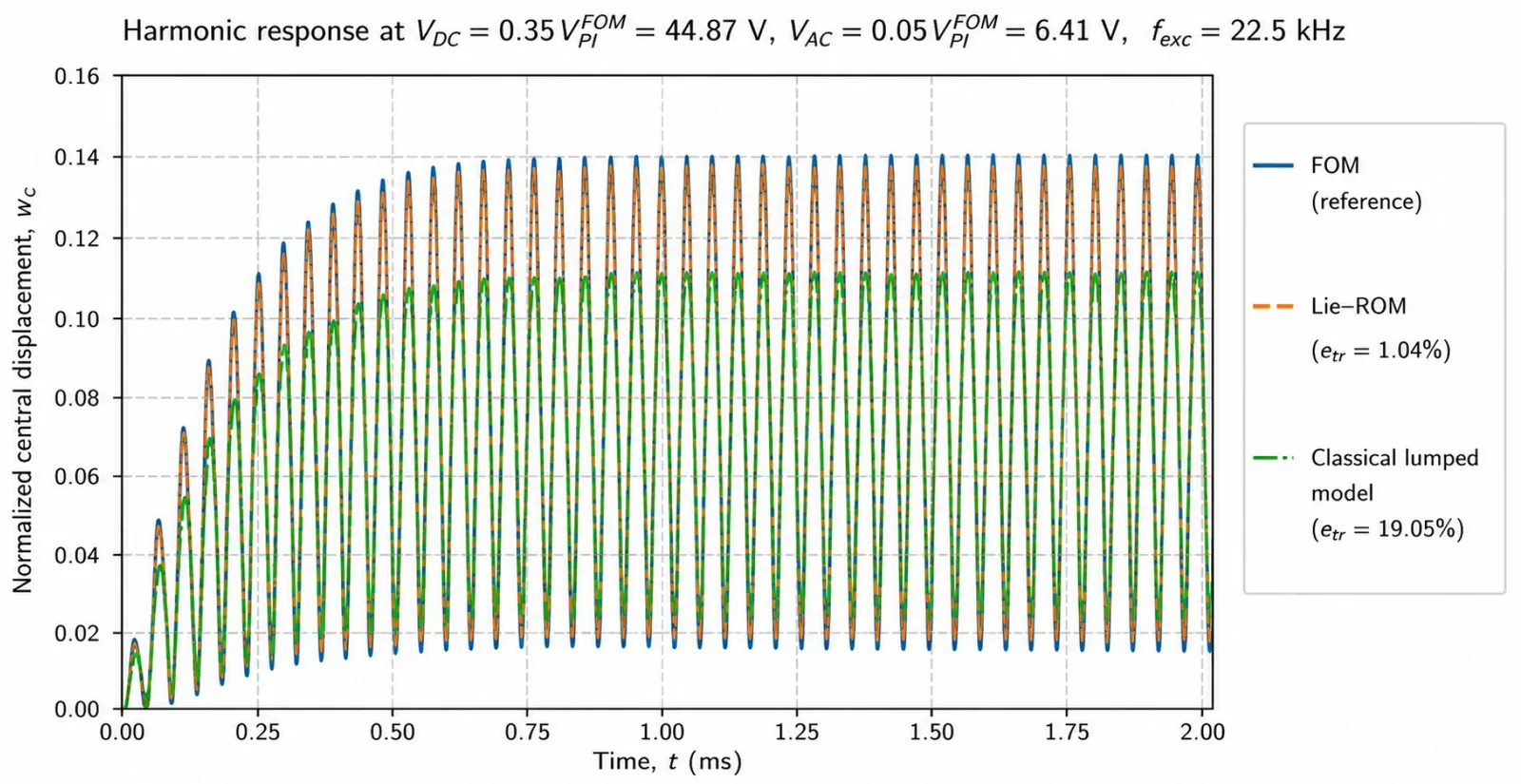
Transient response under harmonic electrostatic actuation ($V_{\mathrm{DC}}=0.35V_{\mathrm{PI}}^{\mathrm{FOM}}$, $V_{\mathrm{AC}}\;=\;0.05V_{\mathrm{PI}}^{\mathrm{FOM}}$, $f_{\mathrm{exc}}=22.5\;\mathrm{kHz}$). Comparison between the continuum model, monomodal Lie-ROM, and classical lumped model. The Lie-ROM model accurately matches the continuum response, whereas the lumped model shows amplitude and phase errors.

The harmonic response was then evaluated for $V\left(t\right)=V_{\mathrm{DC}}+V_{\mathrm{AC}}\sin\left(2\pi f_{\mathrm{exc}}t\right)$ with $V_{\mathrm{DC}}=0.35V_{\mathrm{PI}}^{\mathrm{FOM}}$, $V_{\mathrm{AC}}=0.05V_{\mathrm{PI}}^{\mathrm{FOM}}$, and $f_{\mathrm{exc}}=22.5\;\mathrm{kHz}$, selected near the first biased natural frequency while remaining within the admissible regime before pull-in. Figure 7 shows that (28) closely reproduces the transient and periodic responses of model (9), including the mean displacement, oscillation amplitude, phase, and weak higher-harmonic contribution induced by the nonlinear electrostatic force with $e_{\mathrm{tr}}=1.04\%$. Model (31) instead underestimates the steady-state amplitude and exhibits a phase discrepancy, yielding $e_{\mathrm{tr}}=19.05\%$.

### 8.5. Influence of Fringing Field and Capacitive Feedback

**Figure 8:**
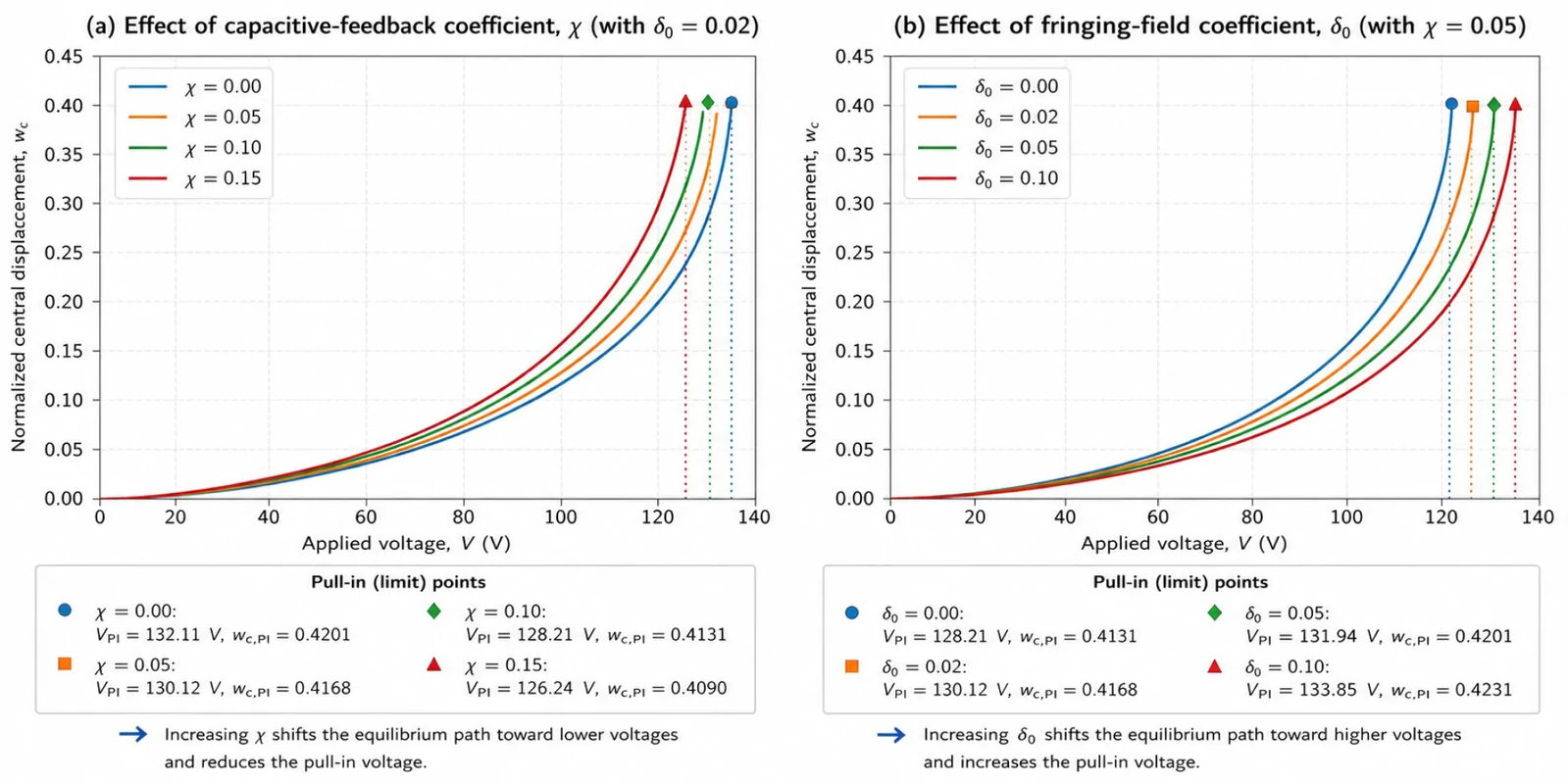
Influence of the electrostatic correction parameters on the static equilibrium response predicted by the monomodal Lie-ROM: (**a**) Effect of the fringing field coefficient $\delta_{0}$ and (**b**) effect of the capacitive feedback coefficient χ. Increasing $\delta_{0}$ promotes pull-in, whereas increasing χ delays the instability.

**Figure 9:**
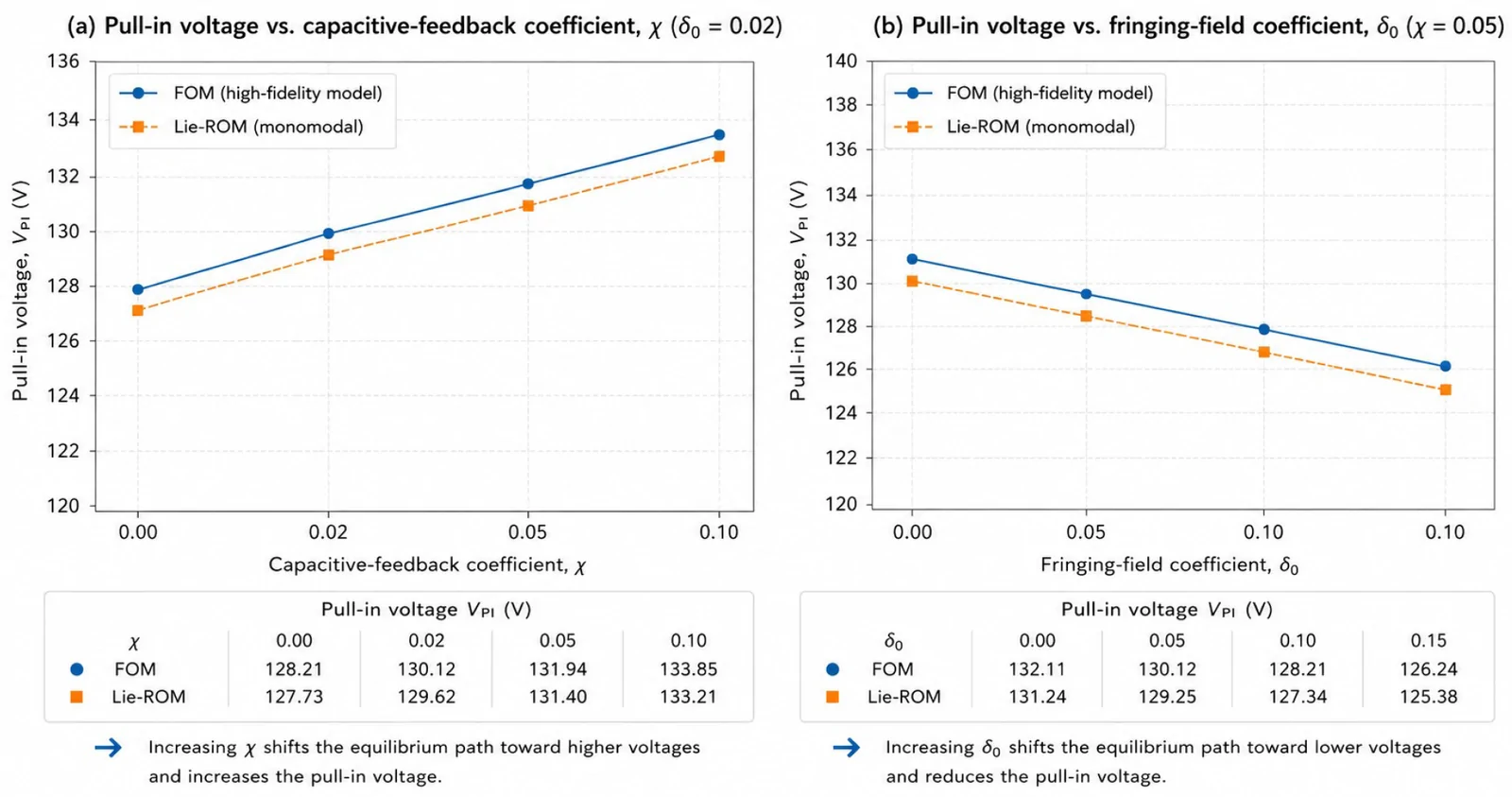
Static pull-in voltage versus (**a**) fringing field coefficient $\delta_{0}$ and (**b**) capacitive feedback coefficient χ. Horizontal lines denote the nominal parameter values.

**Figure 10:**
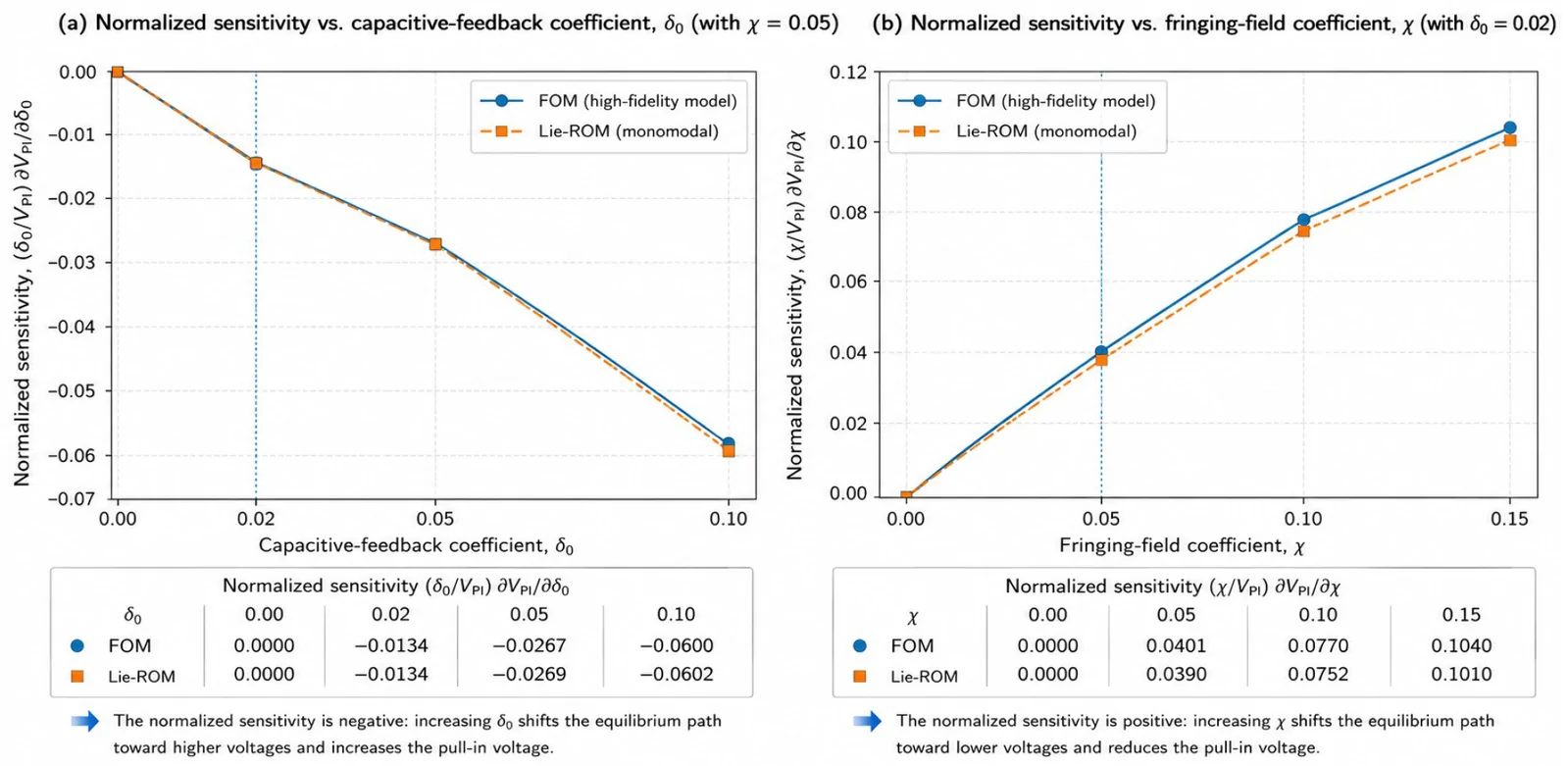
Normalized pull-in-voltage sensitivities with respect to (**a**) $\delta_{0}$ and (**b**) χ. Dotted lines indicate the nominal configuration ($\delta_{0}=0.02$, χ=0.05).

The two electrostatic correction mechanisms retained by the continuum formulation and the proposed Lie-ROM were investigated by varying $0\leq \delta_{0}\leq 0.10$ and 0≤χ≤0.15, with all other parameters fixed at their nominal values. The fringing field and capacitive feedback analyses were performed at χ=0.05 and $\delta_{0}=0.02$, respectively. As shown in the left panel of Figure 8, increasing $\delta_{0}$ enhances the positive electrostatic contribution associated with the local displacement gradient term, thereby increasing the central displacement and shifting the limit point toward lower voltages. Conversely, the right panel of Figure 8 shows that increasing χ shifts the equilibrium branches toward higher voltages, which is because the factor ${\left[1+\chi I_{2}\right]}^{2}$ in the electrostatic force denominator reduces the effective electrostatic load. The corresponding pull-in trends are shown in Figure 9: increasing $\delta_{0}$ decreases $V_{\mathrm{PI}}$ (left panel of Figure 9), whereas increasing χ raises it (right panel of Figure 9). Specifically, the FOM pull-in voltage decreases from 130.05V at $\delta_{0}=0$ to 121.70V at $\delta_{0}=0.10$, while increasing χ from zero to 0.15 raises it from 122.90V to 138.20V. Table 12 and Table 13 confirm that the Lie-ROM model reproduces both FOM trends with relative pull-in-voltage errors below 0.4% throughout the investigated ranges. Local sensitivities were evaluated at the nominal configuration χ=0.05 and $\delta_{0}=0.02$. The FOM yields $\frac{\partial V_{\mathrm{PI}}}{\partial \delta_{0}}≃-89.64\;V$ and $\frac{\partial V_{\mathrm{PI}}}{\partial \chi }≃104.38\;V$, corresponding to $S_{\delta_{0}}≃-0.0140$ and $S_{\chi }≃0.0407$, respectively. The Lie-ROM predicts nearly identical values, confirming preservation of the parametric dependence of both electrostatic mechanisms. Figure 10 illustrates the negative sensitivity to $\delta_{0}$ in the left panel of Figure 10 and the positive sensitivity to χ in the right panel of Figure 10; at the nominal configuration, the normalized sensitivities indicate the greater relative influence of capacitive feedback on the pull-in threshold.

### 8.6. Influence of Stretching, Pre-Stress and Dielectric Inhomogeneity

**Figure 11:**
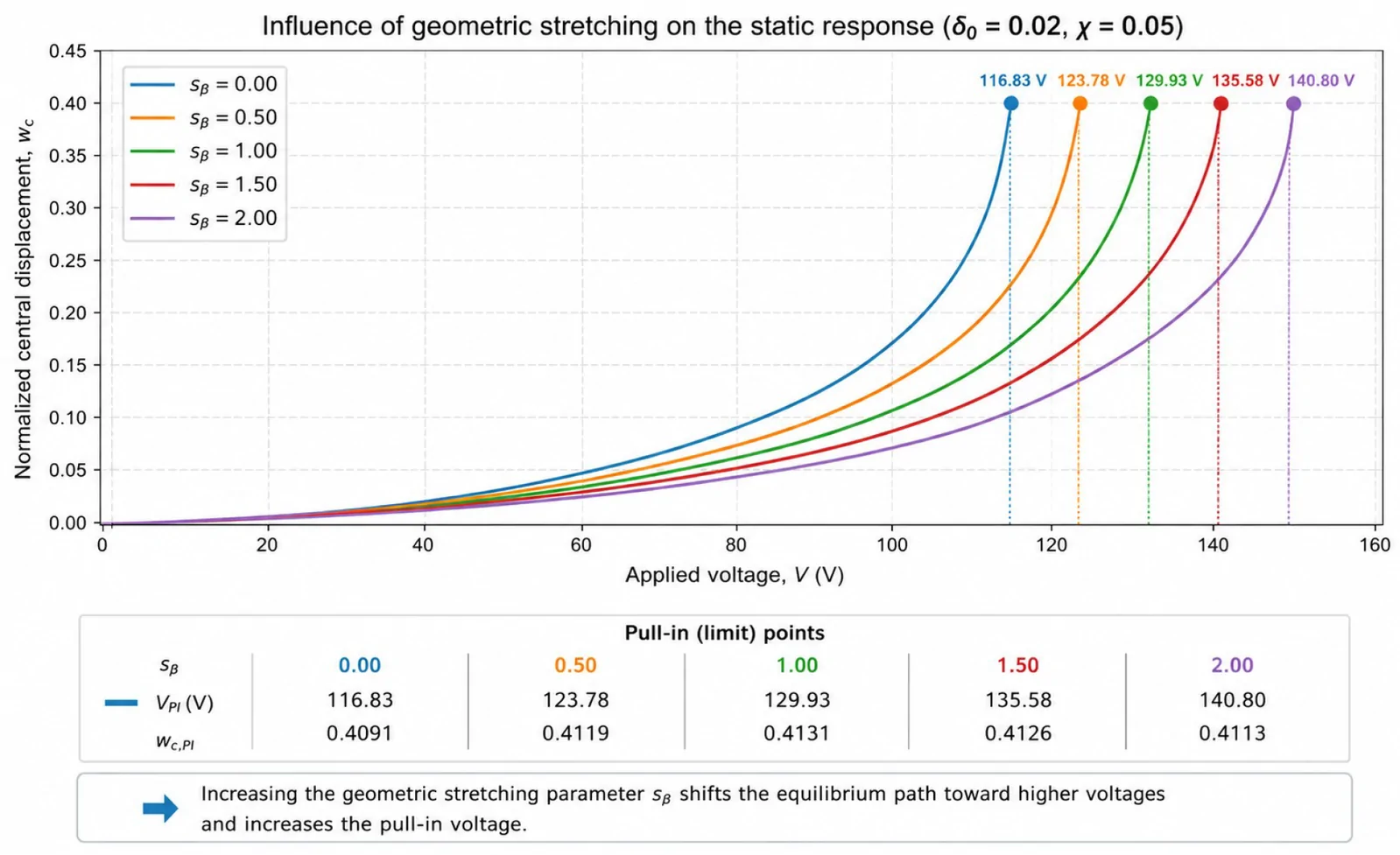
Static equilibrium branches for different stretching coefficients $s_{\beta }=\beta /\beta_{0}$. Larger $s_{\beta }$ increases membrane stiffening, reduces the normalized central deflection, and delays pull-in.

**Figure 12:**
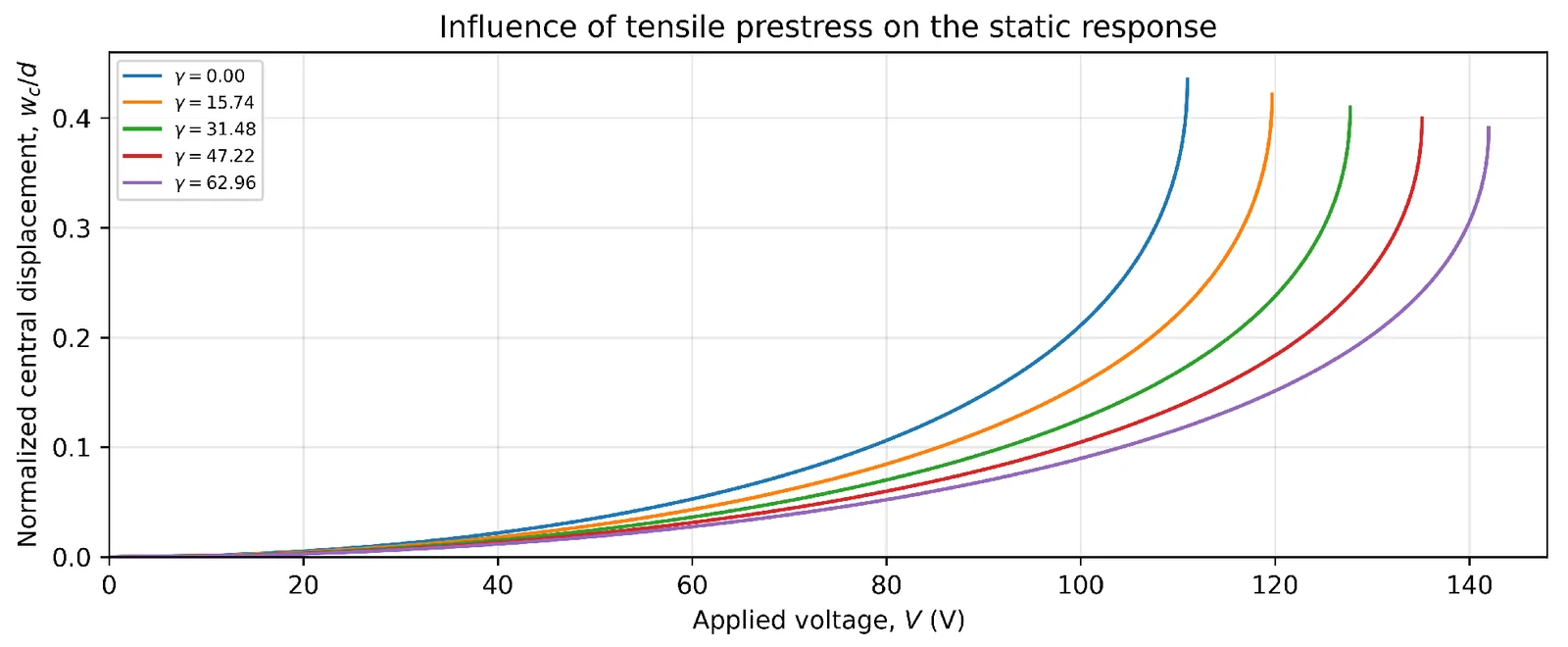
Influence of the dimensionless tensile pre-stress coefficient γ on the static response of the MEMS microplate.

**Figure 13:**
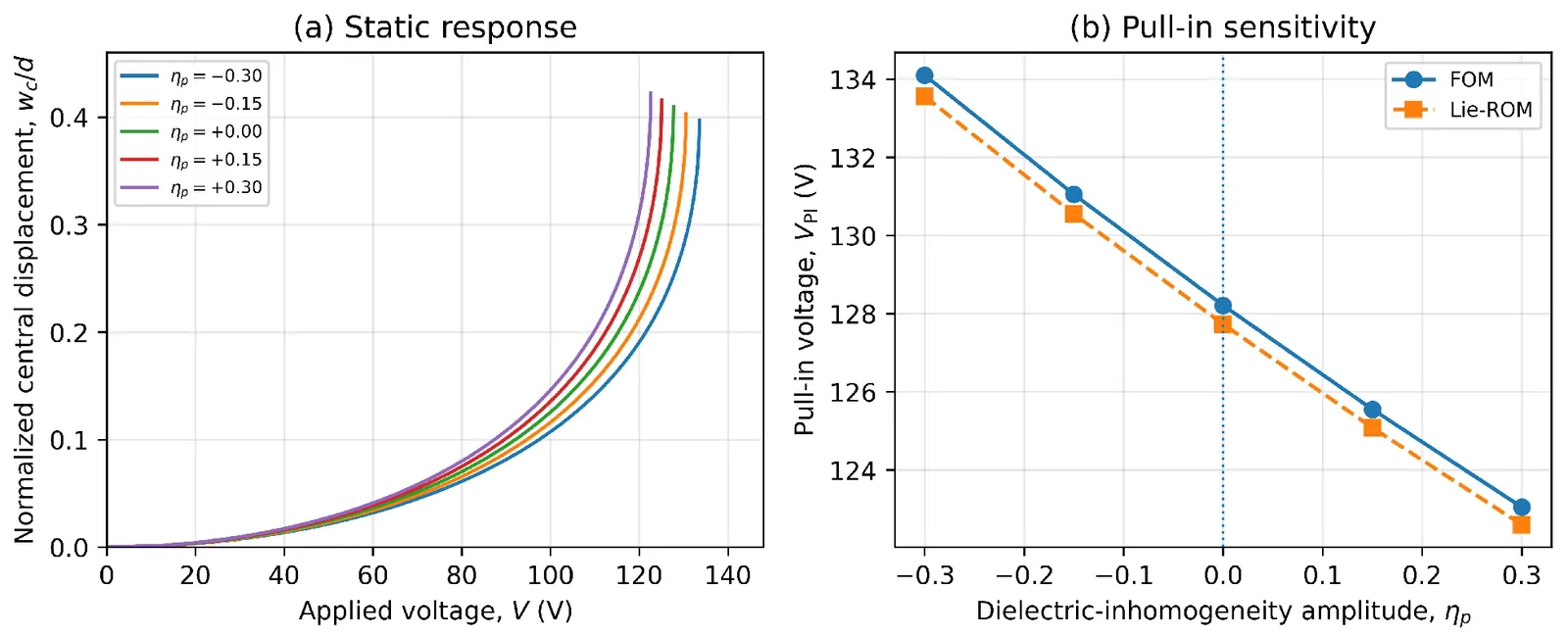
Influence of the dielectric inhomogeneity amplitude $\eta_{p}$ on the static equilibrium response predicted by the proposed Lie-ROM. Positive $\eta_{p}$ decreases the pull-in voltage, whereas negative $\eta_{p}$ increases it.

**Figure 14:**
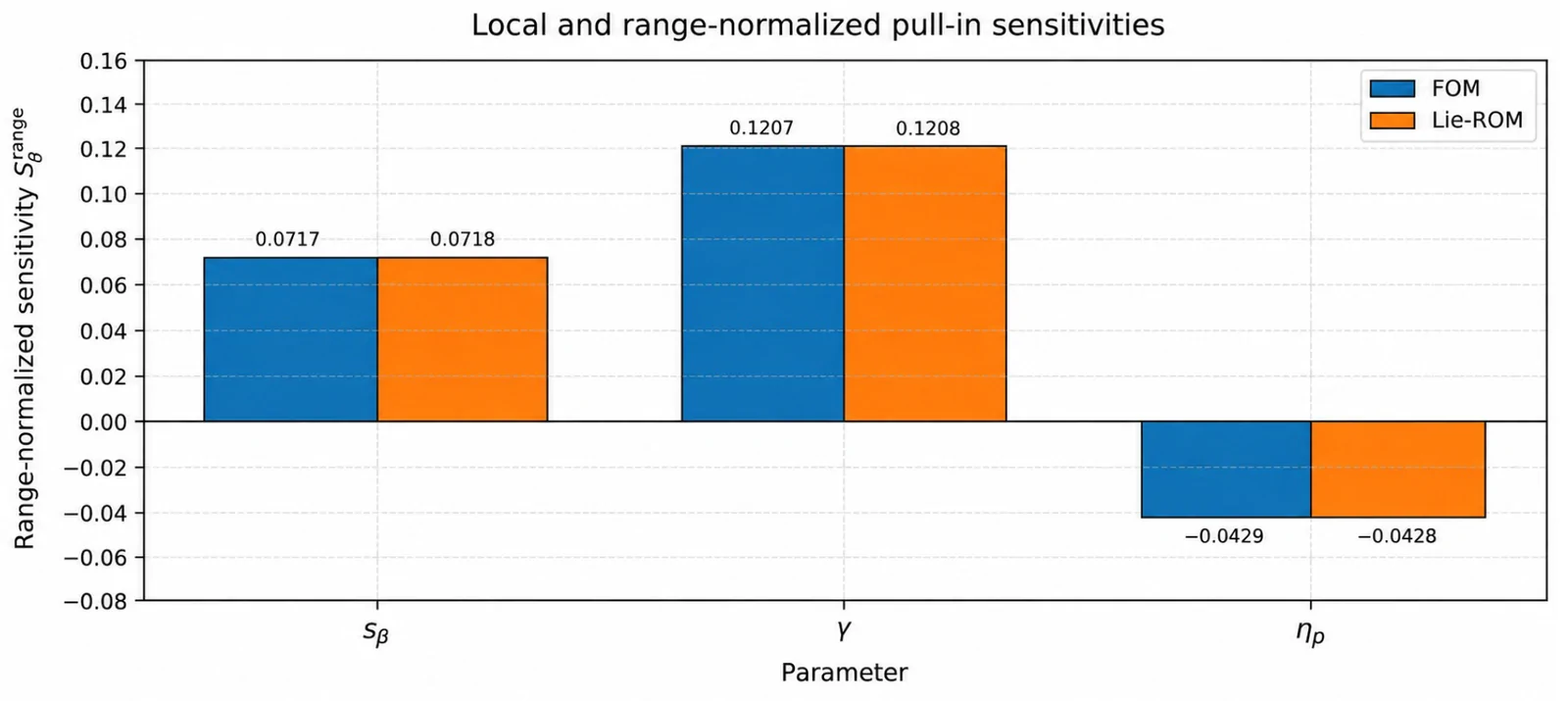
Comparison of the range-normalized pull-in-voltage sensitivities obtained with the FOM and the Lie-ROM for geometric stretching $s_{\beta }$, pre-stress γ, and dielectric inhomogeneity $\eta_{p}$. The range-normalized sensitivity is defined as $S_{\theta }^{\mathrm{range}}=\left(\Delta \theta /V_{\mathrm{PI},0}\right)\left(\partial V_{\mathrm{PI}}/\partial \theta \right)$, where Δθ is the half-width of the investigated parameter interval. The range-based normalization remains well-defined for the nominally zero dielectric inhomogeneity amplitude $\eta_{p,0}=0$. The close agreement between the FOM and Lie-ROM values shows that the reduced formulation accurately reproduces both the magnitude and sign of the local parametric trends over the adopted scaling ranges.

The influence of geometric stretching, residual tensile pre-stress, and spatial dielectric inhomogeneity was investigated by varying one parameter at a time while keeping all others at their nominal values. The stretching coefficient is represented by the normalized multiplier $s_{\beta }=\beta /\beta_{0}$, with $s_{\beta }=1$ denoting the nominal configuration, while the pre-stress coefficient was varied over 0≤γ≤62.96. The dielectric distribution was defined as

(33) $$p\left(\bar{x},\bar{y};\eta_{p}\right)=1+\eta_{p}\cos\left(2\pi \bar{x}\right)\cos\left(\frac{2\pi \bar{y}}{b/a}\right),\;-0.30\leq \eta_{p}\leq 0.30,$$

which preserves the even–even reflection symmetries of the symmetry-preserving modal space. The three parameters affect the static response through distinct mechanisms. As shown in Figure 11, increasing $s_{\beta }$ enhances nonlinear membrane stiffening, reducing the equilibrium deflection and shifting pull-in toward higher voltages; accordingly, the FOM pull-in voltage increases from 117.85V at $s_{\beta }=0$ to 136.50V at $s_{\beta }=2$. Figure 12 shows a similar stabilizing effect of tensile pre-stress through increased initial in-plane stiffness, with the FOM pull-in voltage rising from 111.40V at γ=0 to 142.50V at γ=62.96. At the nominal value γ=31.48, the FOM and Lie-ROM predict 128.21V and 127.73V, respectively. Unlike the pre-stress, which acts even at small deflections, geometric stretching becomes increasingly significant with transverse displacement. As shown in Figure 13, dielectric inhomogeneity modifies the spatial electrostatic loading without altering the structural stiffness: negative $\eta_{p}$ reduces the electrostatic contribution in the central region and delays pull-in, whereas positive $\eta_{p}$ concentrates the loading where displacement is largest and anticipates instability. Consequently, the FOM pull-in voltage decreases from 134.10V at $\eta_{p}=-0.30$ to 123.05V at $\eta_{p}=0.30$. The quantitative results are reported in Table 14. The three parameters exhibit distinct effects on the pull-in voltage. Because the nominal dielectric inhomogeneity amplitude is $\eta_{p,0}=0$, the conventional normalized sensitivity $S_{\theta }=\left(\theta_{0}/V_{\mathrm{PI},0}\right)\left(\partial V_{\mathrm{PI}}/\partial \theta \right)$ is not informative for $\eta_{p}$. Therefore, local parametric sensitivity is characterized using the derivative $\partial V_{\mathrm{PI}}/\partial \theta$ together with a range-normalized sensitivity $S_{\theta }^{\mathrm{range}}=\left(\Delta \theta /V_{\mathrm{PI},0}\right)\left(\partial V_{\mathrm{PI}}/\partial \theta \right)$, where Δθ denotes the half-width of the investigated parameter interval. This definition remains well-posed for parameters that are nominally zero and permits comparison on the basis of the parameter variations considered in the present study. The corresponding sensitivities are compared graphically in Figure 14 and summarized in Table 15. The sensitivity analysis confirms that dielectric inhomogeneity, pre-stress, and geometric stretching produce appreciable but physically distinct changes in the pull-in voltage. The close agreement in both magnitude and sign between the FOM and Lie-ROM sensitivities shows that the reduced formulation accurately reproduces these parametric trends.

### 8.7. Symmetry Preservation and Comparative Unrestricted-Basis Assessment

**Figure 15:**
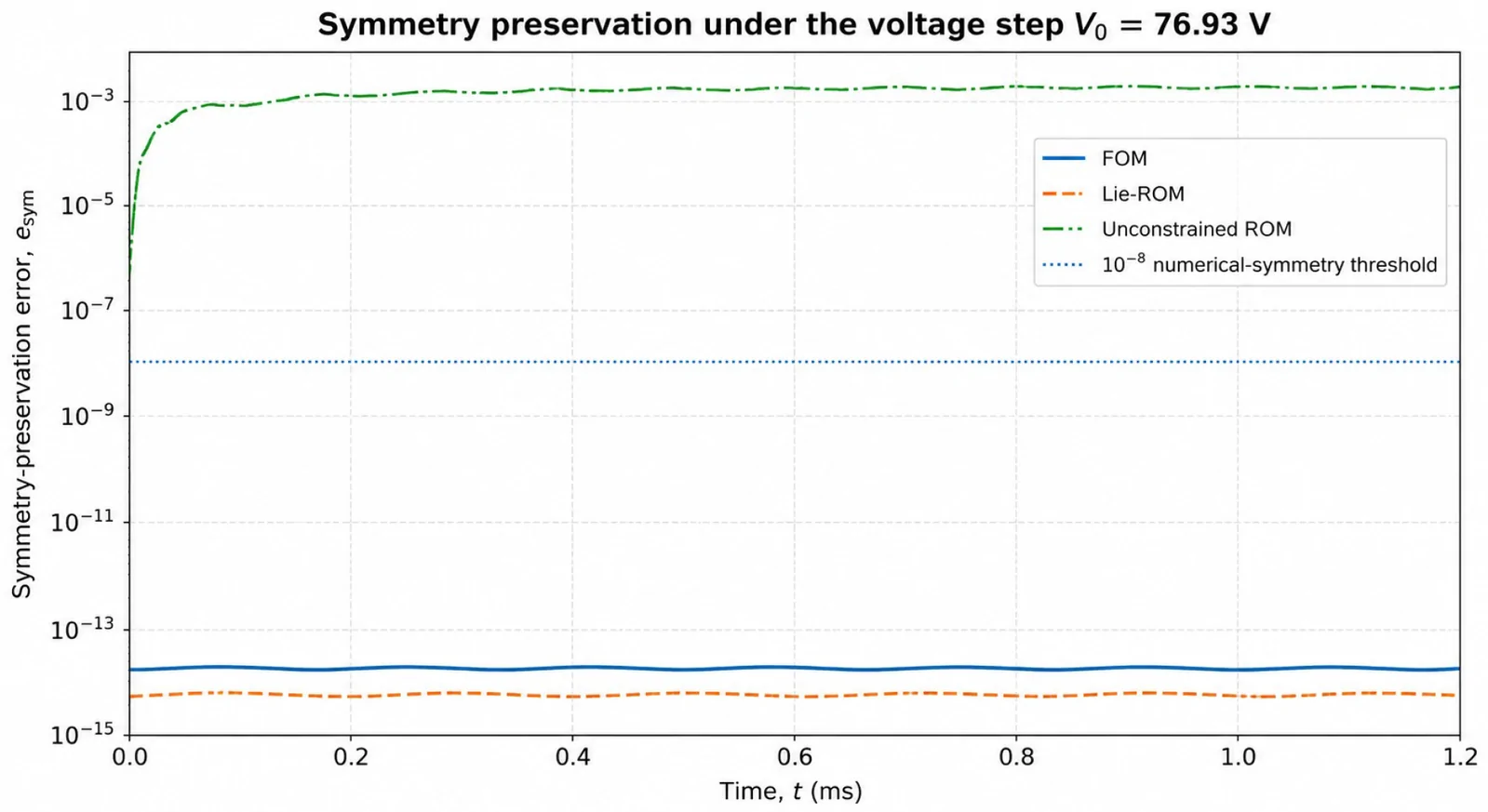
Time evolution of the symmetry preservation error for $V_{0}=0.60V_{\mathrm{PI}}^{\mathrm{FOM}}$. The FOM and Lie-ROM both preserve reflection symmetry, unlike the unconstrained ROM. The dotted line marks the $10^{-8}$ threshold.

**Figure 16:**
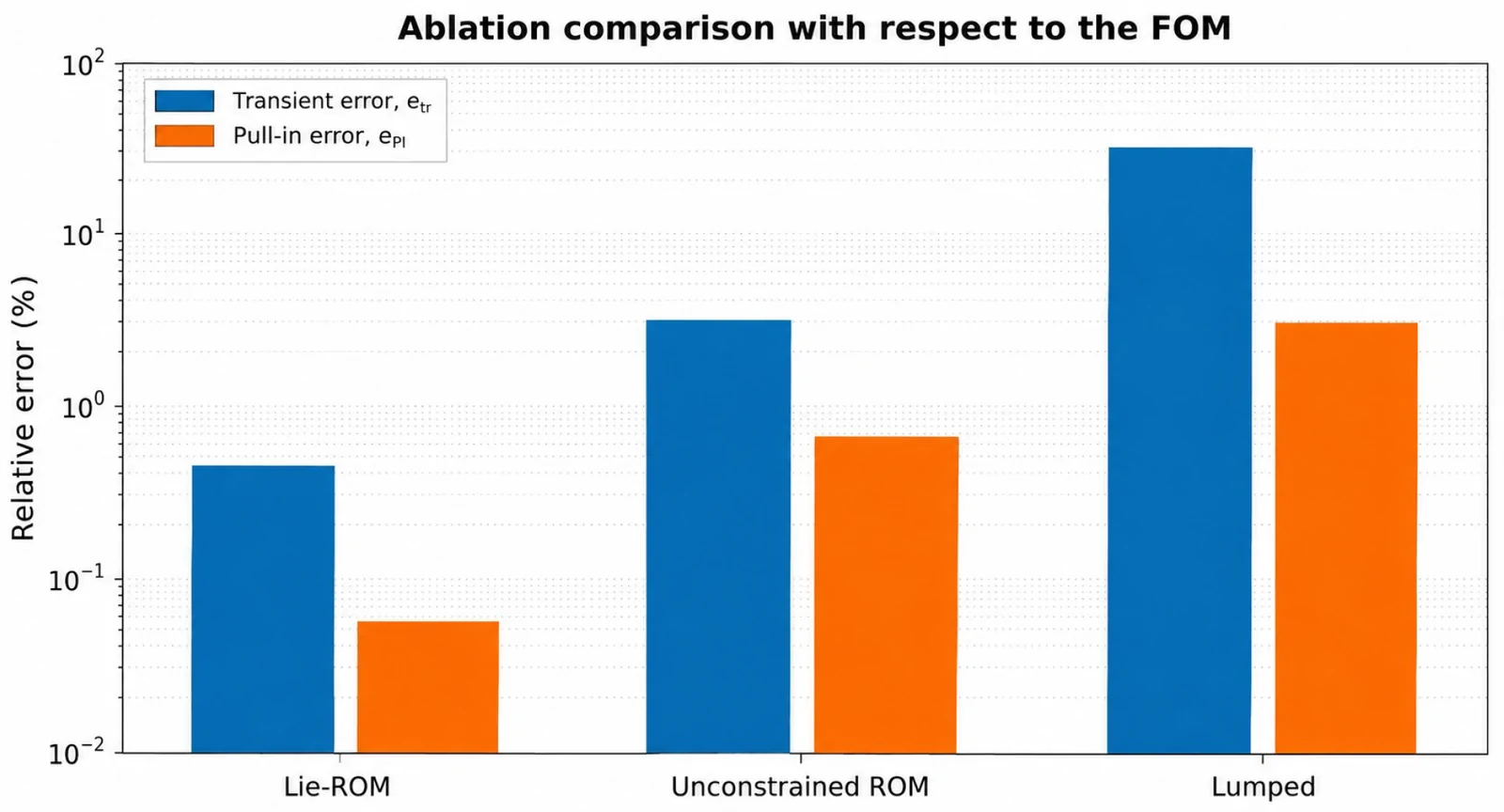
Comparative assessment of transient and pull-in errors relative to the FOM.

At $0.60V_{\mathrm{PI}}^{\mathrm{FOM}}=76.93\;V$, using the nominal physical parameters and zero initial conditions, the symmetry-preservation properties of the proposed reduced-order formulation are assessed against the FOM and an unrestricted-basis Galerkin ROM. The proposed monomodal ROM is constructed from the fundamental clamped biharmonic eigenmode belonging to the even–even invariant subspace, whereas the unrestricted ROM is constructed from the first three eigenmodes of the same discrete clamped biharmonic operator, ordered by eigenvalue and retained without parity filtering. The same modal normalization, quadrature rules, electromechanical formulation, numerical tolerances, and time integration settings are adopted in both Galerkin models. The parity properties of the retained eigenmodes are characterized with respect to the reflection operators $R_{x}$ and $R_{y}$. This classification identifies the symmetry sector associated with each mode and determines whether mixed-parity components are present in the unrestricted modal basis. Figure 15 compares the reflection symmetry error of the FOM and the reduced models. The FOM preserves the prescribed reflection symmetries throughout the simulation, with a maximum error of approximately $2.8\times 10^{-14}$. This value is well below the adopted numerical symmetry threshold of $10^{-8}$, and is consistent with floating-point round-off and numerical quadrature. The monomodal symmetry-preserving ROM exhibits a maximum symmetry error of approximately $8.9\times 10^{-15}$, which is because its reconstructed displacement field belongs identically to the even–even modal subspace. By contrast, the three-dimensional unrestricted-basis ROM exhibits a maximum symmetry error of approximately $1.65\times 10^{-3}$. This result demonstrates that the symmetry-restricted construction preserves the prescribed reflection invariance to machine precision, whereas the unrestricted numerical approximation does not provide the same structural guarantee. The numerical performance of the models is summarized in Figure 16 and Table 16. The proposed single-DoF symmetry-preserving ROM yields a transient response error of $e_{\mathrm{tr}}=0.79\%$ and a pull-in voltage error of $e_{\mathrm{PI}}=0.37\%$. The three-DoF unrestricted-basis ROM gives $e_{\mathrm{tr}}=4.86\%$ and $e_{\mathrm{PI}}=1.77\%$. These results show that for the present benchmark, the symmetry-preserving monomodal approximation is both more accurate and lower-dimensional than the considered unrestricted three-mode approximation; however, because the two ROMs have different numbers of generalized coordinates, this comparison alone does not isolate the effect of parity filtering from that of modal space selection, and should not be interpreted as a controlled demonstration that symmetry restriction necessarily improves numerical accuracy relative to the FOM at fixed reduced dimension. For engineering reference, the classical lumped-parameter model yields $e_{\mathrm{tr}}=25.18\%$ and $e_{\mathrm{PI}}=6.04\%$. Unlike the distributed Galerkin models, the lumped approximation neither reconstructs the full displacement field nor retains the complete distributed and nonlocal electromechanical coupling. It is included as an engineering benchmark rather than as a direct symmetry ablation comparator, and its reflection symmetry indicator is not applicable. The computational results further illustrate the efficiency of the reduced formulation. The proposed monomodal model reduces the 9801 FOM grid values to a single generalized coordinate and requires 0.083s for the reference transient benchmark, compared with 27.9s for the FOM. Thus, the symmetry-preserving model combines substantial dimensional compression, preservation of machine precision on the part of the prescribed reflection invariance, and sub-percent errors in both transient response and pull-in prediction. The present comparison establishes two distinct results: first, restricting the approximation to the even–even invariant subspace guarantees preservation of the prescribed discrete reflection symmetry by construction; second, the resulting monomodal model exhibits lower errors than the considered three-mode unrestricted ROM for the present benchmark. A strict assessment of whether the accuracy improvement originates specifically from symmetry-compatible modal selection, independently of reduced dimension, would require the symmetry-preserving and unrestricted ROMs to be compared at the same number of generalized coordinates.

### 8.8. Robustness Under Parameter Variations

The robustness of model (28) was assessed under simultaneous variations of the principal mechanical, geometric, electrostatic, and damping parameters, namely, Young’s modulus, plate thickness, initial electrode gap, geometric stretching coefficient, residual pre-stress, capacitive feedback coefficient, $E_{\mathrm{ff}}$ coefficient, dielectric inhomogeneity amplitude, and quality factor. A total of 1000 parameter configurations were generated by Latin hypercube sampling (LHS), assuming independent uniform marginal distributions over the ranges reported in Table 17. Sampling was performed using the MATLAB lhsdesign routine with the fixed random seed rng(1,’twister’) to ensure reproducibility. The resulting ensemble was evaluated consistently with the FOM (9), Lie-ROM (28), and classical lumped model while keeping all quantities not included in the sampling at their nominal values.

**Figure 17:**
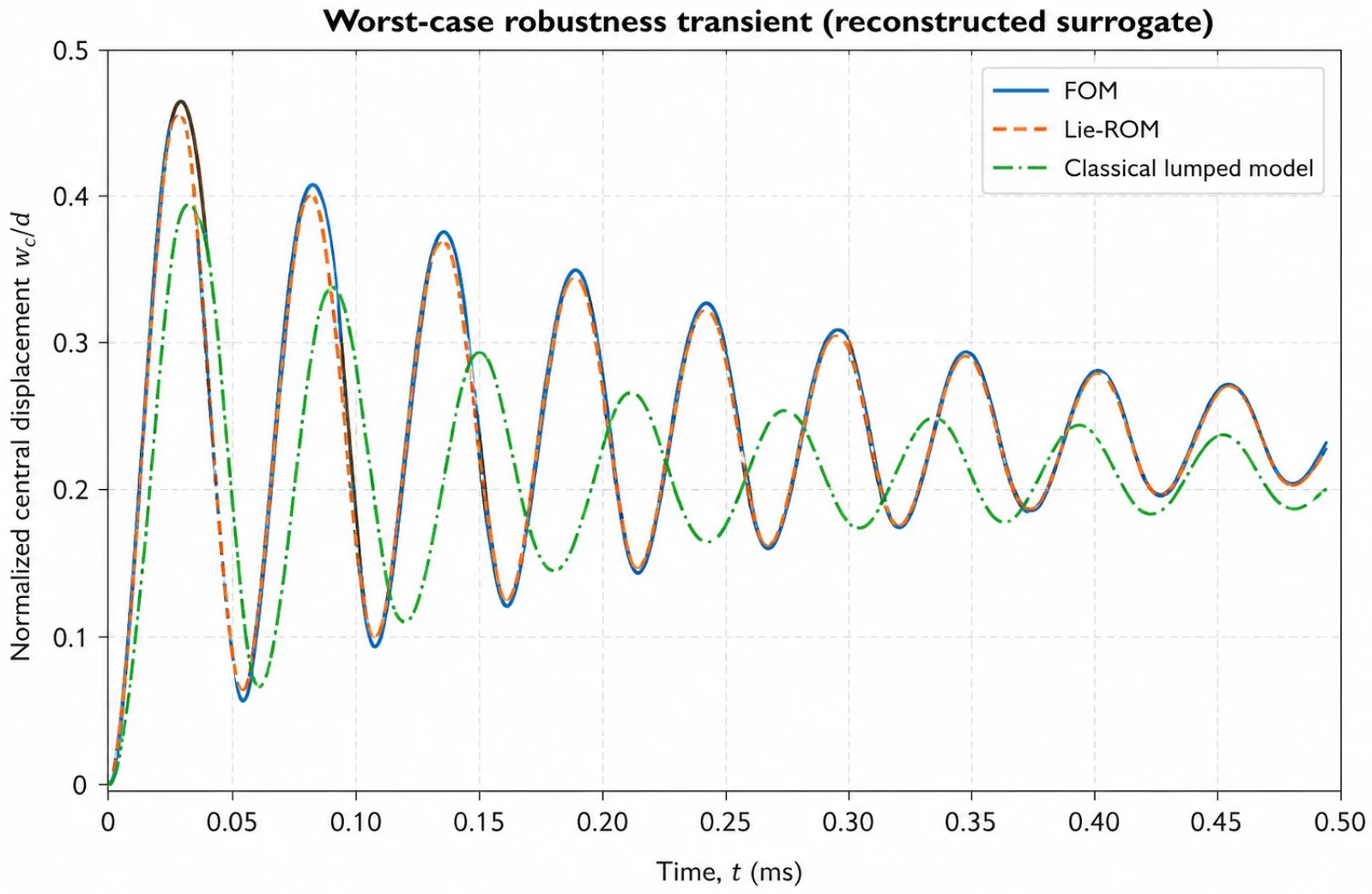
Central displacement histories for the worst-case robustness sample. The Lie-ROM accurately reproduces the FOM, outperforming the classical lumped model.

The statistical robustness indicators are summarized in Table 18. Over the complete parameter ensemble, the Lie-ROM yields an average pull-in voltage error of approximately 0.39% with a 95th percentile below 0.45% and a maximum close to 0.51%, indicating that its agreement with the FOM is preserved under simultaneous variations of the investigated parameters. The model in (28) yields an average pull-in-voltage error of approximately 0.39% with a 95th percentile below 0.45%, while the normalized transient response error has an average value close to 1.3% and remains below 2% over the complete ensemble. By contrast, the model in (31) exhibits average pull-in and transient errors of approximately 6% and 28%, respectively. Figure 17 shows the transient response for the realization producing the largest Lie-ROM transient error. Even in this worst-case configuration, (28) closely reproduces the amplitude, phase, and decay rate predicted by (9), whereas (31) exhibits appreciable discrepancies in amplitude and oscillation frequency. The nonlinear solution procedures also remain robust, with (9) and (28) both converging for 99.8% of the parameter realizations, with mean Newton iteration counts of 5.17 and 3.60, respectively, and no observed violations of the admissibility condition $1-\Phi^{T}\left(x\right)q>0$. The model in (28) maintains sub-percent pull-in discrepancies and low transient response errors relative to the FOM under simultaneous variations of the investigated parameters, demonstrating the robustness of the reduced approximation within the prescribed numerical parameter domain.

### 8.9. Computational Efficiency and Break-Even Analysis

**Figure 18:**
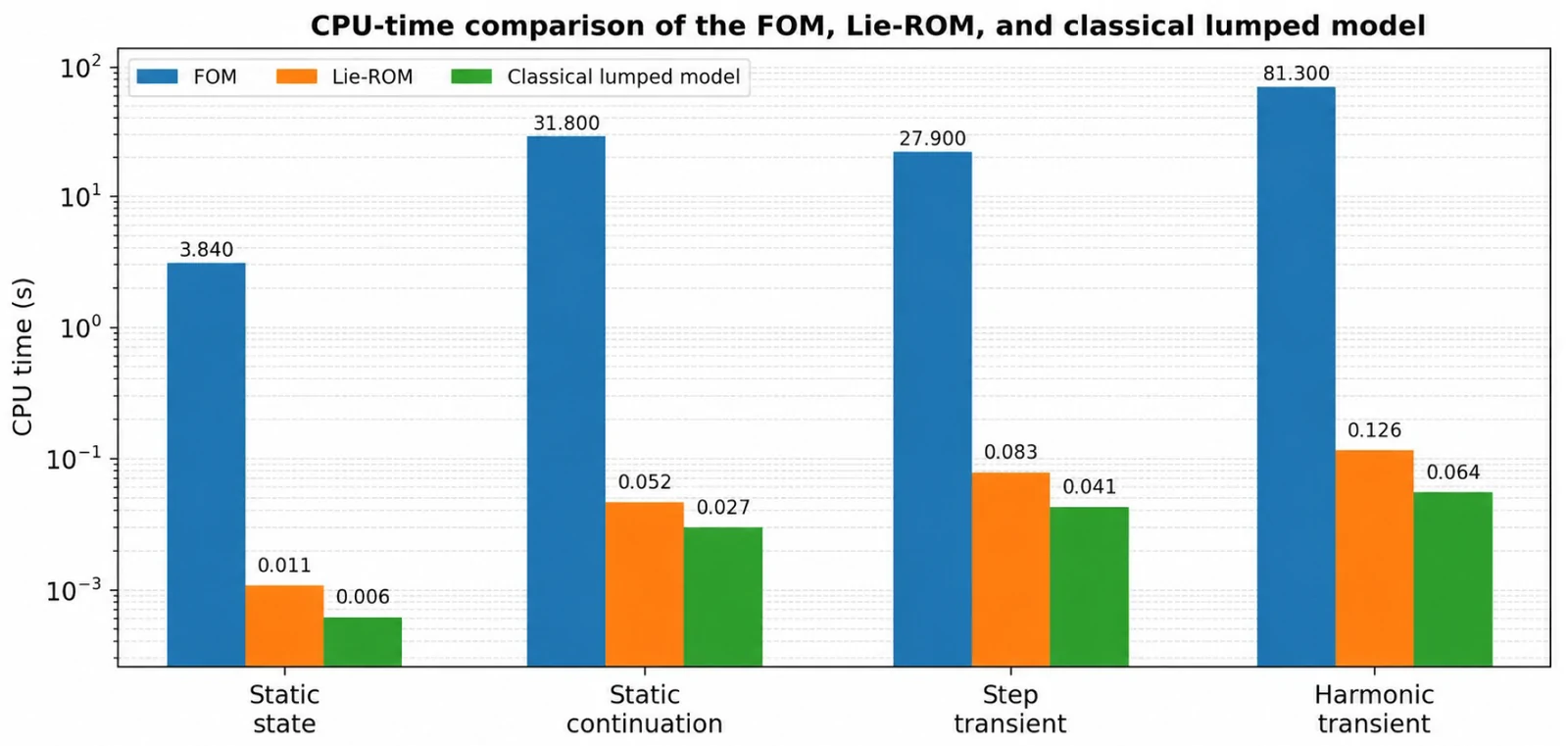
CPU time comparison of the FOM, proposed Lie-ROM, and classical lumped model for the reference numerical benchmarks (logarithmic scale).

As summarized in Figure 18 and Table 19, the proposed Lie-ROM provides a substantial reduction in computational cost relative to the FOM across all the considered numerical benchmarks. The adopted FOM discretization contains $N_{\mathrm{FOM}}=9801$ DoFs, whereas both the monomodal Lie-ROM and the classical lumped model require a single generalized coordinate, yielding a compression ratio of $C_{\mathrm{DOF}}=9801$. Across the considered benchmarks, the Lie-ROM achieves CPU speed-ups ranging from 336.1 to 645.2, reducing the online computational cost by more than two orders of magnitude while retaining the distributed nonlinear electromechanical mechanisms inherited from the continuum formulation. The one-time offline construction of the Lie-ROM requires $T_{\mathrm{off}}=72.4\;s$, including computation and normalization of the retained mode, parity verification, assembly of the reduced coefficients, and initialization of the analytical tangent operators. As reported in Table 20, this cost is recovered after three repeated static continuation analyses, three step-transient simulations, or a single harmonic transient simulation. The reported CPU speed-ups quantify the computational advantage of the Lie-ROM relative to the FOM under the adopted hardware and numerical implementation, but do not establish strict real-time performance, which would require verification against application-specific timing constraints including solver, communication, and hardware-in-the-loop latencies. Accordingly, the present results support the potential of the Lie-ROM for fast, near-real-time, and control-oriented computations rather than demonstrating guaranteed real-time operation.

### 8.10. Limitations of the Present Numerical Verification

Although this section provides an extensive numerical assessment of the proposed methodology, the verification remains within the physical and mathematical assumptions of model (9). The analysis is restricted to the device considered here and to the regime prior to pull-in under Kirchhoff–Love plate theory with moderately large elastic deflections. Alternative geometries and phenomena such as plasticity, fracture, fatigue, adhesion, stiction, dielectric charging, electrostatic trapping, and other effects subsequent to pull-in are outside the scope of the present study. Robustness is assessed through the Latin hypercube sampling analysis reported above, with simultaneous parameter variations over prescribed ranges. This analysis evaluates the robustness of the ROM–FOM agreement within the investigated parameter domain, and should not be interpreted as probabilistic uncertainty quantification. In particular, the adopted sampling distributions are not calibrated from experimental manufacturing tolerances or statistical distributions of material and environmental properties; hence, stochastic manufacturing variability, experimentally characterized uncertainties, environmental effects, and aging are not explicitly quantified. The monomodal model in (28) is sufficient for the reference device, loading conditions, and parameter ranges considered here. More complex geometries, asymmetric or localized forcing, general loading histories, or significant higher-mode contributions may require higher-dimensional symmetry-compatible reduced spaces, which can be constructed within the same symmetry classification framework by including additional modes from the appropriate invariant subspaces. Finally, the present assessment is entirely numerical: the Lie-ROM is assessed against the high-fidelity continuum model (9), and for the engineering comparison, against the classical lumped-parameter model (31). Therefore, the reported agreement constitutes numerical verification of the reduced formulation within the assumptions of the adopted continuum model rather than experimental validation of a fabricated MEMS device. Within these limitations, the results show that the Lie-ROM accurately reproduces the reference continuum response while substantially reducing computational cost. On this basis, the proposed framework provides a basis for future experimental validation, probabilistic uncertainty quantification, multiphysics extensions, higher-dimensional symmetry-compatible reductions, control-oriented applications, and digital twin implementations.

## 9. Conclusions and Perspectives

This work has developed a physics-informed reduced-order modeling framework for nonlinear electrostatically actuated MEMS by combining continuous Lie symmetry classification with symmetry-compatible Galerkin projection. The continuous analysis identifies the transformations compatible with the complete boundary value problem and the restrictions imposed by the fixed geometry, clamped boundary conditions, nonlocal couplings, constitutive coefficients, and electrostatic singularity. For the generic bounded configuration, nontrivial continuous spatial symmetries are suppressed, whereas the surviving discrete reflections define invariant parity subspaces for the reduced-order construction. The resulting Lie-ROM retains the symmetry structure and dominant distributed electromechanical mechanisms of the continuum formulation within a low-dimensional model. Numerical verification against the high-fidelity continuum model (FOM) demonstrates close agreement with the FOM for static equilibria, transient dynamics, pull-in behavior, parametric trends, and reflection symmetry. For the nominal configuration, the monomodal Lie-ROM predicts a pull-in voltage of 127.73V, compared with 128.21V for the FOM, corresponding to an absolute relative error of 0.37% and a signed error of −0.37%. The modal convergence analysis confirms that the fundamental even–even mode captures the dominant response of the reference microplate, while comparison with the classical lumped-parameter model highlights the importance of retaining the distributed electromechanical mechanisms. The symmetry-compatible construction also preserves the prescribed reflection invariances by construction. For the investigated benchmark, the monomodal Lie-ROM is more accurate than the considered three-mode unrestricted approximation; however, because the two models have different dimensions, this comparison does not constitute a controlled fixed-dimensional ablation and does not isolate symmetry restriction as the sole source of the accuracy improvement. The simultaneous parameter variation study further shows that the Lie-ROM remains accurate over the investigated mechanical, geometric, electrostatic, and damping ranges. These results characterize the numerical robustness of the ROM–FOM agreement over the prescribed parameter domain, as opposed to physical predictive accuracy or probabilistic uncertainty quantification based on experimentally calibrated parameter distributions. From a computational perspective, the offline–online formulation provides substantial online savings and a small break-even number, making the reduced model suitable for repeated simulations, sensitivity analysis, optimization, inverse identification, and control-oriented applications. The present results are limited to the fully clamped rectangular microplate, the pre-contact elastic regime, and the assumptions of the adopted continuum formulation. The present formulation is intentionally restricted to the pre-contact regime, w<1, in which a positive electrode gap is maintained and the electrostatic forcing remains finite. Consequently, the current FOM and Lie-ROM are not intended to describe the dynamics after pull-in, electrode impact, or subsequent adhesion. Extending the framework to the regime after pull-in would require replacing the present gap positivity constraint with an appropriate contact formulation and introducing additional physical mechanisms such as unilateral contact forces, impact/contact dissipation, and short-range surface interactions responsible for adhesion and stiction. Such extensions would lead to a nonsmooth or piecewise-smooth electromechanical problem, and would require a corresponding reassessment of both the admissible symmetries and the invariant reduced subspaces used in the Lie-ROM construction. Developing such a symmetry-consistent reduced-order formulation for contact, release, and stiction dynamics represents a natural direction for future work. The present formulation is intended as a reduced electromechanical description of the pre-contact regime and retains the mechanisms explicitly represented in the reference continuum model, including distributed viscous damping, geometric stretching, pre-stress, dielectric inhomogeneity, capacitive feedback, and fringing field effects. However, it does not resolve additional multiphysics mechanisms such as thermoelastic coupling, genuinely nonlinear or state-dependent dissipation, or time-dependent dielectric charging. This distinction is relevant when extrapolating the present results to practical operating conditions. Thermoelastic effects may alter the mechanical response through temperature-dependent stiffness and thermally induced stresses, and may introduce additional thermoelastic dissipation; consequently, temperature variations may affect resonance characteristics and stability margins. The damping term $c\left(x,t\right)w_{t}$ already permits spatially and temporally varying viscous dissipation, but does not represent dissipation laws that depend nonlinearly on velocity, displacement, or vibration amplitude. When such mechanisms become significant, transient decay, oscillation amplitude, phase, and dynamic stability thresholds may differ from those predicted by the present model. Likewise, the prescribed weighting p(x) accounts for spatial dielectric inhomogeneity and its effect on the electrostatic loading, but does not describe the accumulation and relaxation of trapped charge. In devices affected by dielectric charging, the resulting time-dependent modification of the electrostatic actuation may produce bias drift and corresponding changes in equilibrium and stability thresholds. Therefore, the present predictions should be interpreted within the electromechanical mechanisms and parameter ranges explicitly considered here; quantitative application to operating regimes dominated by thermal variations, nonlinear dissipation, or charge accumulation would require an augmented multiphysics formulation and a corresponding reassessment of its symmetry structure before constructing the reduced-order model. Accordingly, the reported agreement constitutes numerical verification against the FOM rather than experimental validation of a fabricated MEMS device. More complex geometries, asymmetric or localized forcing, general loading histories, and responses involving significant higher-mode contributions may require higher-dimensional symmetry-compatible reduced spaces and a new classification of the continuous and discrete symmetries associated with the corresponding geometry and boundary conditions. Although the present Lie classification is derived for a fully clamped rectangular microplate, the proposed methodology is not intrinsically restricted to rectangular domains. Its extension to other MEMS geometries requires reformulating the spatial differential operators and boundary conditions on the corresponding domain and subsequently repeating the Lie invariance analysis for the complete boundary value problem. For example, for a circular membrane or plate, the governing electromechanical formulation can be expressed in polar coordinates, replacing the Cartesian Laplacian and biharmonic operators with their polar counterparts. In the axisymmetric case, the displacement reduces to a radial field w=w(r,t), and rotational invariance provides the natural symmetry structure from which symmetry-compatible modal subspaces can be constructed. The nonlinear electrostatic gap dependence and fringing field contribution retain the same physical roles, while the geometry enters through the radial differential operators, integration measure, and boundary conditions. Therefore, the Lie-classification/Galerkin workflow remains conceptually unchanged, although the resulting symmetry group and admissible basis functions must be recomputed for the circular domain. Perforated membranes constitute a more geometry-dependent extension, as the holes introduce internal boundaries and may break some of the symmetries of the unperforated structure. In that case, the Lie analysis must be performed on the perforated domain together with the boundary conditions imposed on both the external boundary and the hole boundaries. Any residual discrete or continuous symmetry of the complete perforated geometry can then be used to identify invariant subspaces and construct a corresponding symmetry-compatible ROM. Thus, while the proposed framework is transferable to other standard MEMS geometries, the symmetry classification itself is specific to particular geometry and boundary conditions. Future work will address experimental validation on fabricated MEMS devices, probabilistic uncertainty quantification based on experimentally characterized parameter distributions, and higher-dimensional reductions for complex loading conditions and higher-order dynamics. Further developments will include multiphysics effects such as thermoelastic coupling, dielectric charging, nonlinear dissipation, surface interactions, post-contact dynamics, and manufacturing variability. Subject to application-specific timing requirements and implementation constraints, these extensions may further support the use of the proposed framework in parameter estimation, model predictive control, virtual sensing, rapid engineering design, and fast or potentially near-real-time digital twin applications.

## Figures and Tables

**Table 1:** Reference dimensional and normalized parameters of the phosphorus-doped LPCVD polysilicon MEMS microplate.

Quantity	Symbol	Reference Value/Definition	Normalized Value/Model Parameter
Plate length	*a*	300μm	1
Plate width	*b*	200μm	0.6667
Plate thickness	*h*	2.0μm	$6.667\times 10^{-3}$
Initial electrode gap	*d*	2.0μm	$6.667\times 10^{-3}$
Young’s modulus	*E*	163.1GPa	1
Poisson ratio	ν	0.223	0.223
Mass density	ρ	$2330\;\mathrm{kg}\;m^{-3}$	1
Residual tensile stress	$\sigma_{0}$	20.0MPa	represented through $N_{0}$ and γ
Membrane prestress	$N_{0}$	$40.0\;N\;m^{-1}$	represented through γ
Prestress coefficient	γ	$N_{0}a^{2}/D_{p}$	31.48
Nominal stretching coefficient	$\beta_{0}$	$\frac{6}{\left|\Omega \right|}{\left(\frac{d}{h}\right)}^{2}$	9.00
Air-gap relative permittivity	$\varepsilon_{r}$	1.0006	1.0006
Vacuum permittivity	$\varepsilon_{0}$	$8.854\times 10^{-12}\;F\;m^{-1}$	absorbed in normalization
Quality factor	*Q*	50	50
Damping ratio	ζ	1/(2Q)=0.01	0.01
Modal damping coefficient	$c_{0}$	—	$2\zeta \;{\bar{\omega }}_{1}$
Capacitive-feedback coefficient	χ	phenomenological model parameter	0.05
Fringing-field coefficient	$\delta_{0}$	phenomenological model parameter	0.02
Dielectric weighting	*p*	homogeneous reference case	1
Bending stiffness	$D_{p}$	$1.144\times 10^{-7}\;N\;m$	reference value
Normalized bending-stiffness coefficient	$K_{1}$	$K_{1}=D/D_{p}$	1
Operating temperature	$T_{0}$	300K	1

*Notes:* Geometric quantities are normalized with respect to the plate length a=300μm, so that |Ω|=ab=0.6667 in normalized coordinates. Values equal to unity in the last column denote quantities normalized by their corresponding nominal reference values. The dimensionless coefficients γ, $\beta_{0}$, ζ, χ, and $\delta_{0}$ are the parameters entering the normalized electromechanical model. Here, ${\bar{\omega }}_{1}$ denotes the first dimensionless natural angular frequency of the reference configuration. The coefficients χ and $\delta_{0}$ are phenomenological electromechanical parameters adopted for the reference numerical benchmark, and should not be interpreted as intrinsic material properties of LPCVD polysilicon.

**Table 2:** Physical meaning of the operators, coefficients, and nonlinear terms appearing in the governing electromechanical model.

Quantities in (1)	Physical Significance
$\Delta^{2}w\left(x,t\right)$	Kirchhoff–Love biharmonic operator describing microplate bending.
$K_{1}\left(x,t\right)$	Normalized bending stiffness coefficient multiplying the biharmonic operator.
$w_{t}\left(x,t\right)$	Local microplate velocity governing viscous damping.
c(x,t)	Distributed damping coefficient accounting for spatially and temporally varying dissipation.
$w_{tt}\left(x,t\right)$	Inertial term for out-of-plane microplate acceleration.
β	Dimensionless coefficient of membrane stretching
$\int_{\Omega }{\left|\nabla w\left(x,t\right)\right|}^{2}\;dx$	Global geometric stretching functional.
γ	Initial in-plane mechanical pre-stress (residual membrane tension).
Δw(x,t)	Local curvature of the movable microplate governing bending effects.
${\left(\lambda \left(t\right)\right)}^{2}$	Dimensionless electrostatic actuation parameter, proportional to ${\left(V\left(t\right)\right)}^{2}$, measuring electrostatic-to-elastic force ratio.
p(x)	Spatial weighting function describing dielectric inhomogeneity and the distribution of electrostatic loading.
χ	Nonlocal capacitive feedback coefficient.
δ(x,t)	Spatially and temporally varying coefficient weighting the local fringing field contribution to the electrostatic force.
$\frac{{\left(\lambda \left(t\right)\right)}^{2}{\left|\nabla w\left(x,t\right)\right|}^{2}}{{\left(1-w\left(x,t\right)\right)}^{2}}$	Pressure due to $E_{\mathrm{ff}}$ and plate slopes.
$\left[\beta I_{1}\left(t\right)+\gamma \right]\Delta w\left(x,t\right)$	Introduces a stiffness dependent on the global deformation state.
${\left(1-w\left(x,t\right)\right)}^{-2}$	Singularity of the electrostatic force.

**Table 3:** First-order infinitesimal transformations of the extended formulation.

$t^{*}=t+\varepsilon \tau \left(t,x,y,w,A,B\right)+O\left(\varepsilon^{2}\right)$	$x^{*}=x+\varepsilon \xi^{x}\left(t,x,y,w,A,B\right)+O\left(\varepsilon^{2}\right)$
$y^{*}=y+\varepsilon \xi^{y}\left(t,x,y,w,A,B\right)+O\left(\varepsilon^{2}\right)$	$w^{*}=w+\varepsilon \phi \left(t,x,y,w,A,B\right)+O\left(\varepsilon^{2}\right)$
$A^{*}=A+\varepsilon \alpha \left(t,x,y,w,A,B\right)+O\left(\varepsilon^{2}\right)$	$B^{*}=B+\varepsilon \zeta \left(t,x,y,w,A,B\right)+O\left(\varepsilon^{2}\right)$

*Note:* The infinitesimal components α and ζ associated with the auxiliary variables A(t) and B(t) are constrained by preservation of their global time-dependent character and by the integral constraints defining the extended formulation.

**Table 4:** Parity conditions for the constitutive coefficients and initial conditions preserving the reflection symmetries of the rectangular clamped MEMS microplate.

p(−x)=p(x)	p(x,−y)=p(x)	c(−x,t)=c(x,t)	c(x,−y,t)=c(x,t)
δ(−x,t)=δ(x,t)	δ(x,−y,t)=δ(x,t)	$w_{0}\left(-x\right)=w_{0}\left(x\right)$	$w_{0}\left(x,-y\right)=w_{0}\left(x\right)$
$v_{0}\left(-x\right)=v_{0}\left(x\right)$	$v_{0}\left(x,-y\right)=v_{0}\left(x\right)$		

**Table 5:** Continuous Lie symmetry classes for the nonlinear electrostatic MEMS model.

Symmetry Class	Assumptions	Admitted Generators
General class	Arbitrary constitutive coefficients c(x,t), p(x), δ(x,t), λ(t), bounded domain	None
Autonomous class	Time-independent coefficients ($c_{t}=0$, $\delta_{t}=0$, $\dot{\lambda }=0$)	$\partial_{t}$ (time translation)
Spatially homogeneous model on an unbounded domain	Spatially uniform coefficients; absence of physical boundary constraints	$\partial_{x},\;\partial_{y},\;-y\partial_{x}+x\partial_{y}$ (translations and rotation)

**Table 6:** Modal coefficients, reduced nonlinear functionals, and their analytical derivatives defining the one-degree-of-freedom Lie symmetry-preserving reduced-order model (Lie-ROM).

$m=\int_{\Omega }\phi^{2}\;d\Omega$	$c_{\phi }\left(t\right)=\int_{\Omega }c\left(x,t\right)\phi^{2}\;d\Omega$	$k_{b}=\int_{\Omega }{\left(\Delta \phi \right)}^{2}\;d\Omega$
$g=\int_{\Omega }{\left|\nabla \phi \right|}^{2}\;d\Omega$	$I_{1,1}\left(q\right)=gq^{2}$	$B\left(q\right)=\int_{\Omega }\frac{1}{1-q\phi (x)}\;d\Omega$
$P\left(q\right)=\int_{\Omega }\frac{p(x)\phi (x)}{{\left(1-q\phi (x)\right)}^{2}}\;d\Omega$	$D\left(q,t\right)=q^{2}\int_{\Omega }\frac{{\delta \left(x,t\right)\phi \left(x\right)|\nabla \phi \left(x\right)|}^{2}}{{\left(1-q\phi (x)\right)}^{2}}\;d\Omega$	
$B^{\prime }\left(q\right)=\int_{\Omega }\frac{\phi (x)}{{\left(1-q\phi (x)\right)}^{2}}\;d\Omega$	$B^{\prime \prime }\left(q\right)=2\int_{\Omega }\frac{\phi^{2}\left(x\right)}{{\left(1-q\phi (x)\right)}^{3}}\;d\Omega$	$P^{\prime }\left(q\right)=2\int_{\Omega }\frac{p\left(x\right)\phi^{2}\left(x\right)}{{\left(1-q\phi (x)\right)}^{3}}\;d\Omega$
$P^{\prime \prime }\left(q\right)=6\int_{\Omega }\frac{p\left(x\right)\phi^{3}\left(x\right)}{{\left(1-q\phi (x)\right)}^{4}}\;d\Omega$	$J\left(q,t\right)=\int_{\Omega }\frac{{\delta \left(x,t\right)\phi \left(x\right)|\nabla \phi \left(x\right)|}^{2}}{{\left(1-q\phi (x)\right)}^{2}}\;d\Omega$	$D\left(q,t\right)=q^{2}J\left(q,t\right)$
$J_{q}\left(q,t\right)=2\int_{\Omega }\frac{\delta \left(x,t\right)\phi^{2}\left(x\right){\left|\nabla \phi \left(x\right)\right|}^{2}}{{\left(1-q\phi (x)\right)}^{3}}\;d\Omega$		

**Table 7:** Computational environment adopted throughout the numerical campaign.

Item	Specification
Software	MATLAB Release 2026a (MathWorks, Natick, MA, USA)
Operating system	Microsoft Windows 11 Pro (64-bit)
Processor	Intel^®^ Core^TM^ i7-13700K, 16 cores (24 threads), up to 5.4 GHz
Memory	32 GB RAM
Floating-point arithmetic	IEEE 754 double precision
Linear algebra	MATLAB built-in sparse matrix routines
Programming approach	Vectorized modular implementation
Timing protocol	Average over 20 independent runs; initialization, offline preprocessing, and graphical post-processing excluded
Post-processing	MATLAB built-in visualization and data-analysis tools

**Table 8:** Numerical solution settings adopted throughout the computational campaign.

Item	Setting
Static nonlinear solver	Newton–Raphson method with analytical tangent operator
Maximum Newton iterations	30 per voltage increment
Residual convergence criterion	$∥R^{\left(k\right)}{∥}_{2}/{∥R^{\left(0\right)}∥}_{2}<10^{-10}$
Relative solution increment	$∥\Delta {q^{\left(k\right)}∥}_{2}/{∥q^{\left(k\right)}∥}_{2}<10^{-10}$
Continuation strategy	Incremental voltage continuation using the previously converged equilibrium as the initial Newton guess
Initial voltage increment	$\Delta V_{0}=1\;V$
Voltage-increment adaptation	Increment retained when convergence is achieved within five Newton iterations; otherwise successively halved
Near-pull-in criterion	$\lambda_{\min}\;\left[K_{T}\left(V\right)\right]<0.05\;\lambda_{\min}\;\left[K_{T}\left(0\right)\right]$
Near-pull-in voltage increment	ΔV≤0.1V
Minimum voltage increment	$\Delta V_{\min}=10^{-3}\;V$
Pull-in detection	Vanishing of the smallest tangent-stiffness eigenvalue for the FOM; $K_{T}=0$ for the monomodal Lie-ROM
Pull-in refinement tolerance	Successive critical-voltage estimates differing by less than $10^{-3}\;V$
Time-integration solver	MATLAB ode45 adaptive variable-step solver (explicit Dormand–Prince Runge–Kutta scheme)
Relative integration tolerance	$\mathrm{RelTol}=10^{-8}$
Absolute integration tolerance	$\mathrm{AbsTol}=10^{-10}$
Maximum integration step	$\Delta t_{\max}=0.50\;\mu s$
Spatial quadrature	Composite trapezoidal rule using MATLAB trapz along both spatial directions
Admissibility criterion	$1-\Phi^{T}\left(x\right)q>0,\;\forall \;x\in \Omega$
Stopping criterion	Termination when the admissibility condition is violated, the minimum voltage increment is reached without convergence, or the maximum number of Newton iterations is exceeded

**Table 9:** Performance indicators used for comparative assessment of the computational models.

Indicator	Expression	Purpose
Relative central-displacement error	$e_{w_{c}}=\frac{\left|w_{c,\mathrm{model}}-w_{c,\mathrm{FOM}}\right|}{\left|w_{c,\mathrm{FOM}}\right|}\times 100\%$	Accuracy of the static response
Pull-in voltage error	$e_{\mathrm{PI}}=\frac{\left|V_{\mathrm{PI}}^{\mathrm{model}}-V_{\mathrm{PI}}^{\mathrm{FOM}}\right|}{V_{\mathrm{PI}}^{\mathrm{FOM}}}\times 100\%$	Accuracy of pull-in prediction
Signed pull-in voltage error	$e_{\mathrm{PI}}^{\mathrm{sgn}}=\frac{V_{\mathrm{PI}}^{\mathrm{model}}-V_{\mathrm{PI}}^{\mathrm{FOM}}}{V_{\mathrm{PI}}^{\mathrm{FOM}}}\times 100\%$	Direction and magnitude of the pull-in discrepancy
Transient response error	$e_{\mathrm{tr}}=\frac{{\left[\int_{0}^{T}{\left|w_{c,\mathrm{model}}\left(t\right)-w_{c,\mathrm{FOM}}\left(t\right)\right|}^{2}\;dt\right]}^{1/2}}{{\left[\int_{0}^{T}{\left|w_{c,\mathrm{FOM}}\left(t\right)\right|}^{2}\;dt\right]}^{1/2}}\times 100\%$	Accuracy of the dynamic response
CPU speed-up	$S_{\mathrm{CPU}}=\frac{T_{\mathrm{FOM}}}{T_{\mathrm{model}}}$	Computational efficiency
Compression ratio	$C_{\mathrm{DOF}}=\frac{N_{\mathrm{FOM}}}{N_{\mathrm{model}}}$	Reduction in the number of degrees of freedom
Energy-conservation error	$e_{E}=\max_{t\in [0,T]}\frac{\left|E_{\mathrm{model}}\left(t\right)-E_{\mathrm{model}}\left(0\right)\right|}{\left|E_{\mathrm{model}}\left(0\right)\right|}\times 100\%,\;E_{\mathrm{model}}\left(0\right)\neq 0$	Energy conservation in the undamped, unforced case
Symmetry-preservation error	$e_{\mathrm{sym}}=\max_{t\in T_{w}}\max\left\{\frac{{∥w(x,y,t)-w(-x,y,t)∥}_{L^{2}\left(\Omega \right)}}{{∥w(x,y,t)∥}_{L^{2}\left(\Omega \right)}},\;\frac{{∥w(x,y,t)-w(x,-y,t)∥}_{L^{2}\left(\Omega \right)}}{{∥w(x,y,t)∥}_{L^{2}\left(\Omega \right)}}\right\}$	Preservation of the reflection-invariant functional subspace

$w_{c}\left(t\right)=w\left(0,0,t\right)$ denotes the plate center displacement, while FOM denotes the high-fidelity continuum model and “model” denotes either the proposed Lie-ROM model or the classical lumped-parameter model. The relative central displacement error is evaluated only when $|w_{c,\mathrm{FOM}}|>0$. The signed pull-in error $e_{\mathrm{PI}}^{\mathrm{sgn}}<0$ denotes underprediction of the FOM pull-in voltage, whereas $e_{\mathrm{PI}}^{\mathrm{sgn}}>0$ denotes overprediction. The energy conservation error is evaluated for undamped unforced tests with nonzero initial mechanical energy, $E_{\mathrm{model}}\left(0\right)\neq 0$. For the symmetry preservation indicator, $T_{w}={\{t\in \left[0,T\right]:∥w\left(\cdot ,\cdot ,t\right)∥}_{L^{2}\left(\Omega \right)}>0\}$, thereby excluding instants at which the normalized indicator would be undefined. The symmetry preservation indicator applies only to models that reconstruct a distributed displacement field, namely, the FOM and the Lie-ROM; it is not defined for the classical lumped-parameter model.

**Table 10:** Temporal convergence study of the continuum model for a voltage step $V_{0}=0.60V_{\mathrm{PI}}^{\mathrm{FOM}}$. Errors in the peak normalized central displacement are evaluated with respect to the solution obtained with $\Delta t_{\max}=0.25\;\mu s$.

$\Delta t_{\max}$ (μs)	Output Time Points	$\max(w_{c}/d)$	${\left(w_{c}/d\right)}_{\mathrm{ss}}$	Peak Error (%)	CPU Time (s)
2.00	601	0.44218	0.23174	0.745	8.4
1.00	1201	0.43972	0.23129	0.185	14.7
0.50	2401	0.43908	0.23117	0.039	27.9
0.25	4801	0.43891	0.23114	Reference	54.6

**Table 11:** Static pull-in predictions and model dimensions for the reference MEMS microplate. Absolute and signed relative errors are evaluated with respect to the FOM. A negative signed error denotes underprediction of the FOM pull-in voltage, whereas a positive value denotes overprediction.

Model	$V_{\mathrm{PI}}$ (V)	(*w_c_*/*d*)*_PI_*	Abs. Error (%)	Signed Error (%)	Model Dimension
FOM (9)	128.21	0.4131	Reference	0.00	9801 grid values
Lie-ROM (28)	127.73	0.4098	0.37	−0.37	1 generalized coordinate
Lumped model (31)	120.46	0.3365	6.04	−6.04	1 generalized coordinate

*Note:* For the FOM, the reported model dimension corresponds to the 121×81=9801 displacement grid values, including prescribed boundary values for consistency with the compression ratio measure; the clamped boundary values are not treated as independent unknowns.

**Table 12:** Influence of the fringing field coefficient on the static pull-in prediction for χ=0.05. Errors are evaluated with respect to the FOM.

$\delta_{0}$	$V_{\mathrm{PI}}^{\mathrm{FOM}}$ (V)	$V_{\mathrm{PI}}^{\mathrm{Lie}-\mathrm{ROM}}$ (V)	${\left(w_{c}/d\right)}_{\mathrm{PI}}^{\mathrm{FOM}}$	Error (%)
0.00	130.05	129.55	0.4160	0.38
0.02	128.21	127.73	0.4131	0.37
0.05	125.62	125.17	0.4085	0.36
0.10	121.70	121.28	0.4010	0.35

**Table 13:** Influence of the capacitive feedback coefficient on the static pull-in prediction for $\delta_{0}=0.02$. Absolute relative errors are evaluated with respect to the FOM.

χ	$V_{\mathrm{PI}}^{\mathrm{FOM}}$ (V)	$V_{\mathrm{PI}}^{\mathrm{Lie}-\mathrm{ROM}}$ (V)	${\left(w_{c}/d\right)}_{\mathrm{PI}}^{\mathrm{FOM}}$	Abs. Error (%)
0.00	122.90	122.46	0.4040	0.36
0.05	128.21	127.73	0.4131	0.37
0.10	133.35	132.86	0.4210	0.37
0.15	138.20	137.69	0.4280	0.37

**Table 14:** Influence of geometric stretching, pre-stress, and dielectric inhomogeneity on the static pull-in prediction. The stretching multiplier is defined as $s_{\beta }=\beta /\beta_{0}$, with $s_{\beta }=1$ corresponding to the nominal configuration. Absolute relative Lie-ROM errors are evaluated with respect to the FOM.

Parameter	Value	$V_{\mathrm{PI}}^{\mathrm{FOM}}$ (V)	$V_{\mathrm{PI}}^{\mathrm{Lie}-\mathrm{ROM}}$ (V)	Abs. Error (%)
Stretching multiplier $s_{\beta }$	0.00	117.85	117.39	0.39
Stretching multiplier $s_{\beta }$	0.50	123.36	122.89	0.38
Stretching multiplier $s_{\beta }$	1.00	128.21	127.73	0.37
Stretching multiplier $s_{\beta }$	1.50	132.55	132.06	0.37
Stretching multiplier $s_{\beta }$	2.00	136.50	136.00	0.37
Prestress γ	0.00	111.40	110.97	0.39
Prestress γ	15.74	120.15	119.69	0.38
Prestress γ	31.48	128.21	127.73	0.37
Prestress γ	47.22	135.62	135.12	0.37
Prestress γ	62.96	142.50	141.98	0.36
Dielectric amplitude $\eta_{p}$	−0.30	134.10	133.56	0.40
Dielectric amplitude $\eta_{p}$	−0.15	131.05	130.55	0.38
Dielectric amplitude $\eta_{p}$	0.00	128.21	127.73	0.37
Dielectric amplitude $\eta_{p}$	+0.15	125.55	125.08	0.37
Dielectric amplitude $\eta_{p}$	+0.30	123.05	122.60	0.37

**Table 15:** Local and range-normalized pull-in-voltage sensitivities evaluated at the nominal configuration $s_{\beta ,0}=1$, $\gamma_{0}=31.48$, and $\eta_{p,0}=0$. The local sensitivity is defined as $\partial V_{\mathrm{PI}}/\partial \theta$, whereas the range-normalized sensitivity $S_{\theta }^{\mathrm{range}}$ is obtained by multiplying the local sensitivity by the half-width of the investigated parameter interval, Δθ, and dividing the resulting quantity by the nominal pull-in voltage $V_{\mathrm{PI},0}$. The adopted half-widths are $\Delta s_{\beta }=1$, Δγ=31.48, and $\Delta \eta_{p}=0.30$. This range-based normalization remains well-defined for the nominally zero dielectric inhomogeneity amplitude $\eta_{p,0}=0$.

Model	Parameter	$\partial V_{\mathrm{PI}}/\partial \theta$ (V)	Δθ	$S_{\theta }^{\mathrm{range}}$
(9)	$s_{\beta }$	9.19	1.00	0.0717
(9)	γ	0.4914	31.48	0.1207
(9)	$\eta_{p}$	−18.33	0.30	−0.0429
(28)	$s_{\beta }$	9.17	1.00	0.0718
(28)	γ	0.4902	31.48	0.1208
(28)	$\eta_{p}$	−18.23	0.30	−0.0428

**Table 16:** Symmetry-preservation and comparative assessment results for the reference step actuation and static pull-in benchmarks. Errors are evaluated with respect to the continuum model (FOM). The proposed Lie-ROM employs the lowest even–even clamped biharmonic mode, whereas the unrestricted-basis ROM employs the three lowest global modes without parity filtering. The symmetry error is not defined for the lumped model, which does not reconstruct a spatial displacement field. Because the two ROMs have different reduced dimensions, their accuracy comparison is reported as a benchmark assessment rather than as a fixed-dimensional ablation of symmetry-based modal selection.

Model	Basis Selection	$\max e_{\mathrm{sym}}$	$e_{\mathrm{tr}}$ (%)	$e_{\mathrm{PI}}$ (%)	DoF	CPU (s)
FOM	Full spatial discretization	$2.8\times 10^{-14}$	Reference	Reference	9801	27.9
Proposed Lie-ROM	Lowest even–even mode	$8.9\times 10^{-15}$	0.79	0.37	1	0.083
Unrestricted-basis ROM	Three lowest global modes	$1.65\times 10^{-3}$	4.86	1.77	3	0.126
Lumped model (31)	Lumped approximation	–	25.18	6.04	1	0.041

**Table 17:** Parameter ranges adopted for the robustness analysis. Independent uniform marginal distributions were sampled by Latin hypercube sampling using 1000 realizations. Unless otherwise specified, the sampling intervals correspond to ±10% variations about the nominal values; for the nominally zero dielectric inhomogeneity amplitude $\eta_{p}$, the symmetric interval [−0.10,0.10] was adopted.

Parameter	Nominal Value	Sampling Range	Distribution
*E*	163.1GPa	[146.79,179.41]GPa	Uniform
*h*	2.0μm	[1.80,2.20]μm	Uniform
*d*	2.0μm	[1.80,2.20]μm	Uniform
$s_{\beta }$	1.0	[0.90,1.10]	Uniform
γ	31.48	[28.33,34.63]	Uniform
χ	0.05	[0.045,0.055]	Uniform
$\delta_{0}$	0.02	[0.018,0.022]	Uniform
$\eta_{p}$	0	[−0.10,0.10]	Uniform
*Q*	50	[45,55]	Uniform

**Table 18:** Statistical robustness indicators obtained from the 1000-sample Latin hypercube sampling (LHS) ensemble. The FOM is adopted as the reference solution.

Indicator	Mean	Std. dev.	95th Percentile	Maximum
FOM pull-in voltage (V)	128.280	8.512	142.720	149.652
Lie-ROM pull-in voltage (V)	127.783	8.476	142.122	149.169
Lie-ROM pull-in error (%)	0.387	0.036	0.448	0.507
Lie-ROM transient error (%)	1.278	0.179	1.567	1.846
Lumped-model pull-in error (%)	5.955	0.361	6.546	7.051
Lumped-model transient error (%)	27.732	2.203	31.440	33.399

*Note:* Statistical quantities were computed over the successfully converged realizations. The FOM and Lie-ROM converged for 99.8% of the sampled parameter configurations.

**Table 19:** Computational cost, CPU speed-up, and model compression for the reference benchmarks.

Benchmark	FOM (s)	Lie-ROM (s)	Lumped (s)	$S_{\mathrm{CPU}}^{\mathrm{Lie}-\mathrm{ROM}}$	$S_{\mathrm{CPU}}^{\mathrm{lumped}}$
Single static equilibrium	3.840	0.011	0.006	349.1	640.0
Full static continuation	31.800	0.052	0.027	611.5	1177.8
Step transient	27.900	0.083	0.041	336.1	680.5
Harmonic transient	81.300	0.126	0.064	645.2	1270.3

**Table 20:** Offline construction cost and break-even estimates for the proposed monomodal Lie-ROM.

Benchmark	$T_{\mathrm{FOM}}$ (s)	$T_{\mathrm{Lie}-\mathrm{ROM}}$ (s)	$N_{\mathrm{BE}}$
Static continuation	31.800	0.052	3
Step transient	27.900	0.083	3
Harmonic transient	81.300	0.126	1

The one-time offline cost is $T_{\mathrm{off}}=72.4\;s$, including computation and normalization of the mode, parity verification, reduced coefficient assembly, and initialization of the analytical tangent operators. The break-even number $N_{\mathrm{BE}}$ is evaluated by dividing the one-time offline cost $T_{\mathrm{off}}$ by the difference between the FOM and Lie-ROM computational times, $T_{\mathrm{FOM}}-T_{\mathrm{Lie}-\mathrm{ROM}}$, and rounding the resulting value up to the nearest integer.

## Data Availability

No new data were created or analyzed in this study. All results reported in this article were obtained from the mathematical formulation and numerical simulations described in the manuscript.

## Abbreviations

The following abbreviations are used in this manuscript:

BVP	Boundary Value Problem
DoF	Degree of Freedom
MEMS	Micro-Electro-Mechanical Systems
FOM	High-Fidelity Full-Order Model
PDE	Partial Differential Equation
ROM	Reduced Order Model

## Appendix A

**Table A1:** Characteristic scales derived from the reference polysilicon microplate parameters.

Characteristic Quantity	Symbol	Value
Flexural rigidity	$D_{p}$	$1.144\times 10^{-7}\;N\;m$
Surface mass density	ρh	$4.66\times 10^{-3}\;\mathrm{kg}\;m^{-2}$
Total plate mass	$m_{\mathrm{plate}}$	$2.796\times 10^{-10}\;\mathrm{kg}$
Characteristic bending time	$t_{b}$	$1.817\times 10^{-5}\;s$
Characteristic angular frequency	$\omega_{b}$	$5.504\times 10^{4}\;\mathrm{rad}\;s^{-1}$
Characteristic frequency	$f_{b}$	8.760kHz
Characteristic voltage	$V_{b}$	5.049V
Parallel-plate capacitance	$C_{0}$	$2.658\times 10^{-13}\;F$

**Table A2:** Infinitesimal variations of the nonlocal functionals under prolonged Lie transformation. The expressions are written in general transport form; for the physically bounded problem, admissible spatial transformations must additionally preserve the rectangular domain and the associated clamped boundary conditions.

Quantity	Expression
Infinitesimal spatial field	$\xi =\left(\xi^{x},\xi^{y}\right),\;\nabla \;\cdot \;\xi =D_{x}\xi^{x}+D_{y}\xi^{y}.$
Jacobian expansion	$J_{\varepsilon }=1+\varepsilon \nabla \;\cdot \;\xi +O\left(\varepsilon^{2}\right),\;d\Omega^{*}=\left(1+\varepsilon \nabla \;\cdot \;\xi \right)d\Omega +O\left(\varepsilon^{2}\right).$
First prolongation	$\phi^{x}=D_{x}\phi -w_{t}D_{x}\tau -w_{x}D_{x}\xi^{x}-w_{y}D_{x}\xi^{y},$
	$\phi^{y}=D_{y}\phi -w_{t}D_{y}\tau -w_{x}D_{y}\xi^{x}-w_{y}D_{y}\xi^{y}.$
Variation of the stretching integrand	${\delta (|\nabla w|}^{2})=2w_{x}\phi^{x}+2w_{y}\phi^{y}.$
Infinitesimal variation of *A*	$\delta A=\int_{\Omega }\left[2w_{x}\phi^{x}+2w_{y}\phi^{y}+{\left|\nabla w\right|}^{2}\nabla \;\cdot \;\xi \right]\;d\Omega .$
Compatibility coefficient α	$\alpha =\delta A,\;\mathrm{on}\;\mathrm{the}\;\mathrm{constraint}\;\mathrm{manifold}\;G_{1}\left[w,A\right]=0.$
Infinitesimal variation of *B*	$\delta B=\int_{\Omega }\left[\frac{\phi }{{\left(1-w\right)}^{2}}+\frac{\nabla \;\cdot \;\xi }{1-w}\right]\;d\Omega .$
Compatibility coefficient ζ	$\zeta =\delta B,\;\mathrm{on}\;\mathrm{the}\;\mathrm{constraint}\;\mathrm{manifold}\;G_{2}\left[w,B\right]=0.$

**Table A3:** Analytical expressions of the Jacobian matrices and tangent operators associated with the reduced electrostatic and fringing field contributions.

Quantity	Expression
Electrostatic Jacobian	$J_{P}\left(q\right)=\frac{\partial P}{\partial q}=2\int_{\Omega }\frac{p\left(x\right)\;\Phi \left(x\right)\Phi^{T}\left(x\right)}{{\left[1-\Phi^{T}\left(x\right)q\right]}^{3}}\;d\Omega$
Gradient of the capacitive functional	$\nabla_{q}H\left(q\right)=\chi \nabla_{q}I_{2,N}\left(q\right)$
Electrostatic tangent matrix	$\frac{\partial f_{\mathrm{es}}}{\partial q}=\lambda^{2}\left(t\right)\left[\frac{J_{P}\left(q\right)}{H^{2}\left(q\right)}-2\frac{P\left(q\right){\left[\nabla_{q}H\left(q\right)\right]}^{T}}{H^{3}\left(q\right)}\right]$
Fringing-field Jacobian	${\left[J_{D}\left(q,t\right)\right]}_{ij}=\frac{\partial D_{i}}{\partial q_{j}}=$
	2 $\int_{\Omega }\delta \left(x,t\right)\phi_{i}\left(x\right)\left[\frac{\nabla \;\left(\Phi^{T}\left(x\right)q\right)\cdot \nabla \phi_{j}\left(x\right)}{{\left[1-\Phi^{T}\left(x\right)q\right]}^{2}}+\frac{{\left|\nabla \;\left(\Phi^{T}\left(x\right)q\right)\right|}^{2}\phi_{j}\left(x\right)}{{\left[1-\Phi^{T}\left(x\right)q\right]}^{3}}\right]\;d\Omega$
Fringing-field tangent matrix	$\frac{\partial f_{\mathrm{ff}}}{\partial q}=\lambda^{2}\left(t\right)\;J_{D}\left(q,t\right)$

**Table A4:** Independent determination and compatibility conditions used in the kernel Lie classification of the extended electromechanical system. For the generic bounded class, the coefficient functions are treated as arbitrary and the compatibility conditions are combined with the fixed rectangular geometry, clamped boundary conditions, and contact manifold constraint.

Condition Type	Infinitesimal Condition	Consequence
Governing equation	$\phi_{w}=0$	ϕ is independent of *w*
Contact manifold	${\left.\phi \right|}_{w=1}=0$	Together with $\phi_{w}=0$, ϕ≡0
Global character of A(t) and B(t)	$A_{x}=A_{y}=B_{x}=B_{y}=0$, which must be preserved by the infinitesimal transformation	The auxiliary components α and ζ are constrained by the global-variable and integral-constraint structure
Damping coefficient	$\tau c_{t}+\xi_{1}c_{x}+\xi_{2}c_{y}=0$	Compatibility with c(x,y,t); for arbitrary coefficients this restricts $\tau ,\xi_{1},\xi_{2}$
Dielectric weighting	$\xi_{1}p_{x}+\xi_{2}p_{y}=0$	Compatibility with p(x,y); for arbitrary *p*, continuous spatial components are restricted
Fringing-field coefficient	$\tau \delta_{t}+\xi_{1}\delta_{x}+\xi_{2}\delta_{y}=0$	Compatibility with δ(x,y,t)
Electrostatic actuation	$\tau {\left(\lambda^{2}\right)}_{t}=0$	Time translation is admissible only when the actuation is autonomous
Rectangular geometry and clamped boundary conditions	The infinitesimal transformation must preserve x=±a/2, y=±b/2, together with w=0 and $\partial_{n}w=0$ on ∂Ω	Continuous spatial translations and rotations are excluded for the physical bounded device
Generic bounded class	Arbitrary c(x,y,t), p(x,y), δ(x,y,t), and λ(t), subject to the preceding compatibility conditions	No nontrivial continuous generator survives

**Table A5:** Compact compatibility conditions for the infinitesimal components associated with the auxiliary nonlocal variables. The relations hold on the corresponding integral constraint manifolds $G_{1}\left[w,A\right]=0$ and $G_{2}\left[w,B\right]=0$, respectively.

Auxiliary Variable	Compatibility Condition
Stretching functional A(t)	$\alpha =\delta A=\int_{\Omega }\left[2w_{x}\phi^{x}+2w_{y}\phi^{y}+{\left|\nabla w\right|}^{2}\nabla \;\cdot \;\xi \right]\;d\Omega ,\;G_{1}\left[w,A\right]=0$.
Capacitive functional B(t)	$\zeta =\delta B=\int_{\Omega }\left[\frac{\phi }{{\left(1-w\right)}^{2}}+\frac{\nabla \;\cdot \;\xi }{1-w}\right]\;d\Omega ,\;G_{2}\left[w,B\right]=0$.

**Table A6:** Mechanical energy structure of the reduced-order model.

Quantity	Expression
Unforced, undamped mechanical subsystem	$M\ddot{q}+Kq+\beta \left(q^{T}Gq\right)Gq=0.$
Reduced kinetic energy	$T_{N}=\frac{1}{2}{\dot{q}}^{T}M\dot{q}.$
Reduced mechanical potential	$\Pi_{m,N}=\frac{1}{2}q^{T}Kq+\frac{\beta }{4}{\left(q^{T}Gq\right)}^{2}.$
Total mechanical energy	$E_{m,N}=T_{N}+\Pi_{m,N}.$
Mechanical consistency	$\nabla_{q}\Pi_{m,N}=Kq+\beta \left(q^{T}Gq\right)Gq.$
Mechanical energy conservation	For the unforced, undamped mechanical subsystem, $\frac{d}{dt}E_{m,N}=0.$
General energy balance	$\frac{d}{dt}E_{m,N}=-{\dot{q}}^{T}C\left(t\right)\dot{q}+{\dot{q}}^{T}f_{\mathrm{es}}\left(q,t\right)+{\dot{q}}^{T}f_{\mathrm{ff}}\left(q,t\right)+{\dot{q}}^{T}r_{\eta ,N}\left(q,t\right).$
Power interpretation	${\dot{q}}^{T}C\left(t\right)\dot{q}\geq 0$ is the dissipated power, whereas ${\dot{q}}^{T}\left[f_{\mathrm{es}}\left(q,t\right)+f_{\mathrm{ff}}\left(q,t\right)\right]$ is the electromechanical power supplied to the reduced system.
Small-stiffness-perturbation approximation	$r_{\eta ,N}=0\;\mathrm{for}\;\mathrm{the}\;\mathrm{reduced}\;\mathrm{model}\;\mathrm{adopted}\;\mathrm{when}\;\eta \ll 1.$

**Table A7:** Reduced stretching functional and associated matrix representation.

Quantity	Expression
Modal approximation	$w_{N}\left(x,t\right)=\sum_{j=1}^{N}q_{j}\left(t\right)\phi_{j}\left(x\right).$
Gradient expansion	$|\nabla w_{N}{|}^{2}=\sum_{j=1}^{N}\sum_{k=1}^{N}q_{j}q_{k}\;\nabla \phi_{j}\cdot \nabla \phi_{k}.$
Reduced stretching functional	$I_{1,N}\left(q\right)=\sum_{j=1}^{N}\sum_{k=1}^{N}q_{j}q_{k}\int_{\Omega }\nabla \phi_{j}\cdot \nabla \phi_{k}\;d\Omega .$
Reduced stretching matrix	$G_{jk}=\int_{\Omega }\nabla \phi_{j}\cdot \nabla \phi_{k}\;d\Omega .$
Quadratic representation	$I_{1,N}\left(q\right)=q^{T}Gq.$
Gradient	$\nabla_{q}I_{1,N}=2Gq.$
Hessian	$\nabla_{q}^{2}I_{1,N}=2G.$

**Table A8:** First- and second-order derivatives of the reduced capacitive functional $I_{2,N}\left(q\right)$.

Quantity	Expression
Partial derivative	$\frac{\partial I_{2,N}}{\partial q_{j}}=\int_{\Omega }\frac{\phi_{j}\left(x\right)}{{\left(1-\Phi^{T}\left(x\right)q\right)}^{2}}\;d\Omega$, $\nabla_{q}I_{2,N}$ = $\int_{\Omega }\frac{\Phi (x)}{{\left(1-\Phi^{T}\left(x\right)q\right)}^{2}}\;d\Omega$
Hessian components and Matrix	$\frac{\partial^{2}I_{2,N}}{\partial q_{i}\partial q_{j}}=2\int_{\Omega }\frac{\phi_{i}\left(x\right)\phi_{j}\left(x\right)}{{\left(1-\Phi^{T}\left(x\right)q\right)}^{3}}\;d\Omega$, $\nabla_{q}^{2}I_{2,N}=2\int_{\Omega }\frac{\Phi \Phi^{T}}{{\left(1-\Phi^{T}q\right)}^{3}}\;d\Omega$
Convexity property	$\forall z\in R^{N}$, $z^{T}\nabla_{q}^{2}I_{2,N}z=2\int_{\Omega }\frac{{\left(\Phi^{T}z\right)}^{2}}{{\left(1-\Phi^{T}q\right)}^{3}}\;d\Omega \geq 0$, which implies that $I_{2,N}$ is convex on every convex subset of $Q_{N,\varepsilon_{g}}$.

**Table A9:** Compact matrix notation adopted for the reduced-order formulation.

Quantity	Definition
Modal basis vector	$\Phi \left(x\right)={\left[\phi_{1}\left(x\right)\;\cdots \;\phi_{N}\left(x\right)\right]}^{T}.$
Generalized coordinate vector	$q\left(t\right)={\left[q_{1}\left(t\right)\;\cdots \;q_{N}\left(t\right)\right]}^{T}.$
Modal approximation	$w_{N}\left(x,t\right)=\Phi^{T}\left(x\right)q\left(t\right).$
Time derivatives	$w_{N,t}\left(x,t\right)=\Phi^{T}\left(x\right)\dot{q}\left(t\right),\;w_{N,tt}\left(x,t\right)=\Phi^{T}\left(x\right)\ddot{q}\left(t\right).$
Spatial gradient	$\nabla w_{N}\left(x,t\right)=\sum_{j=1}^{N}q_{j}\left(t\right)\nabla \phi_{j}\left(x\right).$
Laplacian	$\Delta w_{N}\left(x,t\right)=\sum_{j=1}^{N}q_{j}\left(t\right)\Delta \phi_{j}\left(x\right).$
Biharmonic operator	$\Delta^{2}w_{N}\left(x,t\right)=\sum_{j=1}^{N}q_{j}\left(t\right)\Delta^{2}\phi_{j}\left(x\right).$

**Table A10:** Structure of the Galerkin residual associated with the modal approximation.

Residual Contribution	Expression
Inertial term	$w_{N,tt}$
Damping term	$c\left(x,t\right)\;w_{N,t}$
Bending term	$\Delta^{2}w_{N}$
Prestress and geometric stretching	$-\left[\beta I_{1,N}\left(q\right)+\gamma \right]\Delta w_{N}$
Electrostatic force	$-\frac{\lambda^{2}\left(t\right)\;p\left(x\right)}{{\left(1-w_{N}\right)}^{2}{\left[1+\chi I_{2,N}\left(q\right)\right]}^{2}}$
Fringing-field contribution	$-\frac{\lambda^{2}\left(t\right)\;\delta \left(x,t\right)\;{\left|\nabla w_{N}\right|}^{2}}{{\left(1-w_{N}\right)}^{2}}$
Galerkin residual	$R_{N}=\sum \left(\mathrm{all}\;\mathrm{previous}\;\mathrm{contributions}\right)$
Galerkin orthogonality	${\langle \phi_{i},R_{N}\rangle }_{L^{2}\left(\Omega \right)}=0,\;i=1,\ldots ,N.$

**Table A11:** Fundamental properties of the reduced mass, damping, bending, and stretching matrices.

Matrix	Quadratic Form	Property
Mass matrix M	$z^{T}Mz=\int_{\Omega }{\left(\sum_{j=1}^{N}z_{j}\phi_{j}\right)}^{2}\;d\Omega ,\;\forall \;z\neq 0$	Positive definite if the modal basis is linearly independent.
Damping matrix C(t)	$z^{T}C\left(t\right)z=\int_{\Omega }c\left(x,t\right){\left(\sum_{j=1}^{N}z_{j}\phi_{j}\right)}^{2}\;d\Omega$	Positive semidefinite whenever c(x,t)≥0 a.e. in Ω.
Bending stiffness matrix $K^{\left(b\right)}$	$z^{T}K^{\left(b\right)}z=\int_{\Omega }{\left[\Delta \left(\sum_{j=1}^{N}z_{j}\phi_{j}\right)\right]}^{2}\;d\Omega ,\;\forall \;z\neq 0$	Positive definite.
Stretching matrix G	$z^{T}Gz=\int_{\Omega }{\left|\nabla \left(\sum_{j=1}^{N}z_{j}\phi_{j}\right)\right|}^{2}\;d\Omega ,\;\forall \;z\neq 0$	Positive definite for the clamped modal basis.

## Appendix B

**Proof of Lemma 1.** The assumed regularity of the displacement field guarantees that all spatial derivatives required by the Kirchhoff–Love plate model are well-defined. Consequently, the global stretching functional remains finite for every time instant. Moreover, restricting the analysis to the regime prior to pull-in ensures that the movable electrode remains uniformly separated from the fixed counter-electrode, meaning that the electrostatic integrand is uniformly bounded over the computational domain and the associated capacitive functional is well-defined. Finally, the temporal continuity of the displacement field implies the continuity of both nonlocal functionals: the stretching functional inherits this property from the continuity of the spatial gradient, whereas the capacitive functional follows from the uniform separation from the electrostatic singularity together with the dominated convergence theorem. Hence, both auxiliary nonlocal functionals belong to C([0,T]), completing the proof. □

**Proof of Proposition 1.** The proof consists of two steps. First, the introduction of the auxiliary functionals $I_{1}\left[w\right]$ and $I_{2}\left[w\right]$ does not introduce any approximation, since these quantities simply replace the corresponding nonlocal integral operators by equivalent global variables. Therefore, for a fixed bending coefficient, the nonlocal structure of the original integro-partial differential equation is reformulated exactly. Second, the bending coefficient is decomposed into a unit reference contribution and a uniformly bounded perturbation. Substitution of this decomposition into the original governing equation separates the reference biharmonic operator from a residual term associated with the stiffness perturbation. Since the displacement field is sufficiently regular and the perturbation is uniformly bounded by η, the residual satisfies ${∥r_{\eta }∥}_{L^{2}\left(\Omega \right)}=O\left(\eta \right)$ provided that the biharmonic contribution remains uniformly bounded with respect to the stiffness perturbation, which is consistent with the assumed regularity of the displacement field. Consequently, retaining the residual yields a formulation that remains equivalent to the original governing equation, whereas neglecting it under the assumption η≪1 leads to the approximate canonical model stated in Proposition 1. Thus, the introduction of the auxiliary nonlocal variables is exact, while the only approximation involved in the canonical model used subsequently is associated with neglecting the small residual generated by the bending coefficient perturbation. □

**Proof of Proposition 2.** Preservation of the fixed-gap contact manifold requires the infinitesimal generator to be tangent to the contact surface. Hence, its vertical component must vanish at the prescribed contact level, which excludes nontrivial constant translations and homogeneous scalings of the displacement variable. However, this tangency condition alone does not imply that the vertical component vanishes throughout the admissible state space. The stronger restriction follows from the Lie invariance conditions of the complete extended electromechanical system. In particular, expanding the fourth-order prolonged invariance condition for the governing equation and splitting it with respect to the independent differential terms yields the determining equation $\phi_{w}=0$; thus, the admissible vertical component is independent of the displacement variable. Combining this result with its required vanishing on the fixed contact manifold implies ϕ≡0. Consequently, no nontrivial continuous infinitesimal transformation of the displacement variable is admissible within the fixed-gap formulation. □
